# Unsupervised home spirometry *versus* supervised clinic spirometry for respiratory disease: a systematic methodology review and meta-analysis

**DOI:** 10.1183/16000617.0248-2022

**Published:** 2023-09-06

**Authors:** Rohan Anand, Rebecca McLeese, John Busby, Jonathan Stewart, Mike Clarke, William D-C. Man, Judy Bradley

**Affiliations:** 1 Wellcome-Wolfson Institute for Experimental Medicine, Queen's University Belfast, Belfast, UK; 2 Centre for Public Health, Queen's University Belfast, Belfast, UK; 3 Royal Brompton and Harefield Hospitals, Guy's and St. Thomas’ NHS Foundation Trust, London, UK; 4 Faculty of Life Sciences and Medicine, King's College London, London, UK; 5 National Heart and Lung Institute, Imperial College London, London, UK

## Abstract

**Background:**

The number of patients completing unsupervised home spirometry has recently increased due to more widely available portable technology and the COVID-19 pandemic, despite a lack of solid evidence to support it. This systematic methodology review and meta-analysis explores quantitative differences in unsupervised spirometry compared with spirometry completed under professional supervision.

**Methods:**

We searched four databases to find studies that directly compared unsupervised home spirometry with supervised clinic spirometry using a quantitative comparison (*e.g.* Bland–Altman). There were no restrictions on clinical condition. The primary outcome was measurement differences in common lung function parameters (forced expiratory volume in 1 s (FEV_1_), forced vital capacity (FVC)), which were pooled to calculate overall mean differences with associated limits of agreement (LoA) and confidence intervals (CI). We used the I^2^ statistic to assess heterogeneity, the Quality Assessment of Diagnostic Accuracy Studies (QUADAS-2) tool to assess risk of bias and the Grading of Recommendations Assessment, Development and Evaluation (GRADE) approach to assess evidence certainty for the meta-analyses. The review has been registered with PROSPERO (CRD42021272816).

**Results:**

3607 records were identified and screened, with 155 full texts assessed for eligibility. We included 28 studies that quantitatively compared spirometry measurements, 17 of which reported a Bland–Altman analysis for FEV_1_ and FVC. Overall, unsupervised spirometry produced lower values than supervised spirometry for both FEV_1_ with wide variability (mean difference −107 mL; LoA= −509, 296; I^2^=95.8%; p<0.001; very low certainty) and FVC (mean difference −184 mL, LoA= −1028, 660; I^2^=96%; p<0.001; very low certainty).

**Conclusions:**

Analysis under the conditions of the included studies indicated that unsupervised spirometry is not interchangeable with supervised spirometry for individual patients owing to variability and underestimation.

## Tweetable abstract

**Unsupervised spirometry for assessing patients with respiratory conditions requires caution as it underestimates spirometry in clinic; clinicians must consider the potential for differences. Home and clinic spirometry measurements are not interchangeable.** https://bit.ly/42pqwG7

## Introduction

Traditionally, patients perform spirometry in a clinic setting supervised by a trained professional under standardised conditions [1, 2]. Spirometry is used extensively for diagnosis and for monitoring patients with respiratory conditions between clinic visits, with measurements used to assess disease severity and control [3–5]. In clinical research, lung function is an important outcome measure to assess intervention efficacy [6–8] and is a constituent of clinical trial core outcome sets for chronic obstructive pulmonary disease (COPD) [9], bronchiectasis [10] and pulmonary infections [11].

The Association for Respiratory Technology & Physiology (ARTP) advise that spirometry should be conducted under the supervision of professionals who have completed comprehensive training [12]. However, accelerated by the COVID-19 pandemic and with increased pressure on healthcare systems, routine respiratory services and clinical trials have now adopted unsupervised remote spirometry mainly for the monitoring of patients [13–15]. These portable spirometers have been advocated by healthcare providers [16–18] and have been positively received by patients [19–22], despite no conclusive evidence that unsupervised and supervised spirometry measurements are equivalent to those obtained in clinic. It is crucial to know whether unsupervised assessments are valid and reliable before mass uptake. However, if feasible, remote unsupervised spirometry could support virtual healthcare services in routine care and enable more pragmatic trial designs [23], such as the development and scaling of decentralised clinical trials [24].

The primary objective of the review was to determine if spirometry measurements completed by patients unsupervised at home are different to those obtained under the supervision of a trained professional. It assessed differences between two quantitative methods of measurement used as part of respiratory care in clinical research and clinical practice, rather than assessing the effects of the care itself [25]. Secondary objectives were to explore adherence to unsupervised spirometry, patient satisfaction/acceptability, technical issues, quality of spirometry data, adverse events and costs.

## Methods

The analysis explicitly focused on measurement differences between two methods, unsupervised and supervised spirometry (definitions in supplementary material), and so was completed as a test accuracy review. The protocol for this review was registered prospectively on PROSPERO (ID: CRD42021272816) [26], providing full details on methods. Variations between the protocol and review are described in the supplementary material.

### Criteria for study inclusion

Studies were included if they compared values obtained from unsupervised spirometry to those obtained from supervised spirometry. These could be from using the same or different spirometers. There was no maximum time difference between measurements. Eligible study designs included cross-sectional, longitudinal, randomised or non-randomised controlled or crossover studies in which participants performed both forms of spirometry. Sub-studies embedded within another study were also eligible. There were no restrictions on the type of publications included but they had to be complete datasets, *i.e.* ongoing studies with preliminary data were not included.

### Criteria for study exclusion

We excluded studies in which there was no comparison group and if the publication was not in English.

### Population, intervention, comparator, outcomes

The population, intervention, comparator, outcomes (PICO) terms are detailed fully in the published protocol [26]. In brief, there were no restrictions based on age, disease type or clinical condition. We deemed the intervention group for this review as unsupervised spirometry in the complete absence of a clinician or other professional support. The comparator group was supervised spirometry use in the presence of a clinician or other relevant professional and could include assistance *via* telephone or video link. The primary outcome was measurement of lung function in the intervention and comparator groups, primarily assessed by mean differences in forced expiratory volume in 1 s (FEV_1_), forced vital capacity (FVC), forced expiratory flow at 25–75% of FVC (FEF_25–75%_) and peak expiratory flow (PEF) measurements. Secondary outcomes included exploring adherence, quality criteria, cost, participant satisfaction/acceptability, technical issues and adverse events.

### Search strategy

We conducted searches for relevant studies from database inception to 15 July 2021 in the electronic databases MEDLINE, Embase, the Cochrane Central Register of Controlled Trials (CENTRAL) and a grey literature search on Open Access Theses and Dissertations (OATD) (no eligible studies were retrieved from the grey literature search). We also checked the reference lists of eligible studies or related reviews for additional studies and did forward citation screening of eligible studies. The full search strategy including Medical Subject Headings (MeSH) terms was validated by a medical librarian. All database searches were completed by one author. Additional search strategy information, including the specific search strategy used for each source, can be found in the supplementary material.

### Selection of studies

Search results were imported into the systematic review manager software, Covidence (www.covidence.org). This platform was used for abstract screening, full-text screening and data extraction of eligible studies. Any two authors independently screened titles and abstracts according to the eligibility criteria. Studies deemed potentially eligible had their full texts independently screened by any two authors. Any disagreements or uncertainties were resolved through discussion and, if needed, the involvement of other authors.

### Data extraction

Data were extracted independently for each included study by two authors using a customised data extraction form developed in Covidence. Information extracted included the type of study, eligibility criteria, participant characteristics and details of the spirometers with associated measurements (mean differences, standard deviations and correlations). Outcomes of interest were FEV_1_, FVC, FEF_25–75%_ and PEF. If any of the required data were not available or insufficient, they were requested from the corresponding author of the study.

### Data synthesis and statistical methods

Mean differences and standard deviations were extracted from studies which reported Bland–Altman analyses. Standard deviations were inferred from confidence intervals and limits of agreement (LoA) when they were not reported directly. When no relevant data were reported we imputed the median standard deviation from all other studies reporting that outcome [27]. Meta-analyses were conducted using DerSimonian–Laird random-effects models and the statistical heterogeneity evaluated using the I^2^ statistic [28] and interpreted using the thresholds defined by the Cochrane Handbook [29]. We calculated pooled mean differences with associated 95% confidence intervals, which represent uncertainty around the mean bias estimate. However, to increase the clinical utility of our results, we also calculated pooled LoA around the mean differences, which reflects an interval within which 95% of the differences would lie for a given measurement. Pooled standard deviations were calculated according to the methods outlined in the Cochrane Handbook [29]. Supervised measurements were subtracted from unsupervised, so a negative mean difference indicated that unsupervised spirometry measurements were lower than supervised measurements. The percentage of results that would be expected to have a difference >200 mL was also calculated.

We grouped diseases as obstructive lung disease (COPD and asthma), interstitial lung disease (idiopathic pulmonary fibrosis, interstitial lung disease and pulmonary sarcoidosis), suppurative lung disease (cystic fibrosis) and transplant (lung transplantation and haematopoietic cell transplantation). We reported results for different clinical groups separately if they were presented within the same study and could be separated [30]. Additionally, we performed prespecified subgroup analysis to investigate heterogeneity by patient age (children *versus* adult) and risk of bias (low risk of bias *versus* high risk of bias). We used the baseline measurement for studies that conducted spirometry at multiple timepoints. Pearson and Spearman correlation coefficients measuring the association between unsupervised and supervised spirometry were extracted and pooled based on the Fisher transformation. Data relating to secondary outcomes are presented descriptively. We collated data if it was documented that spirometry had been conducted according to American Thoracic Society (ATS)/European Respiratory Society (ERS) guidelines [1]. Two-tailed tests were used throughout and the threshold for statistical significance was set to 0.05. All analyses were conducted using STATA version 16 (StataCorp, College Station, TX, USA).

### Risk of bias assessment

We assessed risk of bias of included studies using the Quality Assessment of Diagnostic Accuracy Studies (QUADAS-2) tool, which is the most appropriate method to assess risk of bias in this type of review [31]. It assesses risk across four key domains (D1–D4): patient selection, index test (unsupervised spirometry), reference standard (supervised spirometry) and patient flow/timing. A study was determined to be at an overall risk of bias if one or more domains were judged to be at high or unclear risk of bias. A study was determined to be at an overall low risk if all the following were applied: adequate quality criteria for both the supervised and unsupervised spirometry measures, appropriate patient selection and appropriate time intervals between the two measurements, as per QUADAS guidance. A tailored extraction form for this review was piloted and used (available in the supplementary material). Two review authors independently used the tool to assess eligible studies. Any disagreements or uncertainties were resolved through discussion and, if needed, the involvement of other authors.

### Quality of evidence

We assessed overall certainty of the evidence for the primary outcome using the principles of the Grading of Recommendations Assessment, Development and Evaluation (GRADE) approach specific to diagnostic test accuracy [32, 33].

## Results

### Literature search

**FIGURE 1:**
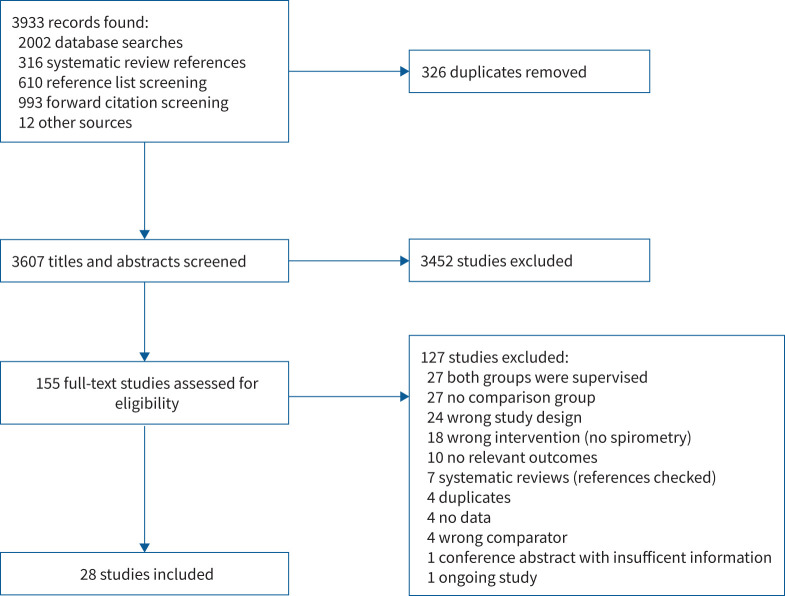
Preferred Reporting Items for Systematic Reviews and Meta-Analyses (PRISMA) flow diagram.

Following database searches, reference list screening and citation screening, 3933 records were identified (figure 1). Following removal of duplicates, 3607 records had titles and abstracts screened and 155 full texts were checked for eligibility. After full-text screening, 28 studies were determined to meet all eligibility criteria and included in the review.

### Study characteristics

Characteristics of included trials are summarised in table 1. This review included 25 prospective studies and three retrospective studies. Included studies totalled 4560 patients, ranging from nine to 2161. Studies included four distinct patient cohorts: interstitial lung disease (n=10) [35, 41, 43–48, 54, 58], transplant (n=8) [36, 38, 42, 49, 51, 56, 57, 59], obstructive airways disease (n=5) [19, 39, 40, 50, 53] and suppurative lung disease (n=5) [30, 34, 37, 52, 55]. In all studies, unsupervised spirometry was completed using a portable handheld spirometer, with a variety of device manufacturers and models used. Eight out of 28 studies [19, 30, 34, 38, 42, 53, 56, 58] explicitly stated that the quality criteria for both unsupervised and supervised measurements was according to ATS/ERS criteria. Five out of 28 studies [19, 34, 53, 57, 58] used the same spirometer for both the unsupervised and supervised measurements.

**TABLE 1:** Characteristics of included studies

**Study**	**Study design**	**Country**	**Mean age (years)**	**Grouping (disease)**	**Patients (n)**	**Device used for unsupervised spirometry**	**Outcomes reported**
**Bell 2022 [34]**	Longitudinal	Australia	37	Suppurative lung disease (CF)	74	Air Next (NuvoAir)	FEV_1_, FVC
**Broos 2018 [35]**	Longitudinal	The Netherlands	43	Interstitial lung disease (pulmonary sarcoidosis)	21	MicroDiary (CareFusion)	FVC
**Cheng 2016 [36]**	Longitudinal	USA	51^#^	Transplant (haematopoietic cell transplantation)	571	KoKo Peak Pro6 (Ferraris Respiratory) or PiKo-6 (Pulmonary Data Services)	FEV_1_, FVC
**Edmondson 2020 [37]**	Single day cross-over	UK and Canada	10^#^	Suppurative lung disease (CF)	67	Lung Monitor BT SMART(Vitalograph)	FEV_1_
**Finkelstein 1993 [38]**	Longitudinal	USA	50	Transplant (lung transplant)	18	Advanced Medical Systems Inc.	FEV_1_, FVC
**Finkelstein 2000 [19]**	Longitudinal	USA	42	Obstructive airways disease (Asthma)	32	V2120 (Vitalograph)	FEV_1_, FVC, FEF_25–75%_, PEF
**Gerzon 2020^¶^ [30]**	Single day cross-over	Netherlands	CF 9 Asthma 10	Suppurative lung disease (CF) and obstructive airways disease (asthma)	CF 36 Asthma 81	AM2+ (CareFusion)	FEV_1_
**Huang 2021 [39]**	Longitudinal	UK	41	Obstructive airways disease (asthma)	12	mSpirometer (Cohero Health)	FEV_1_
**Kerwin 2019^¶^ [40]**	Longitudinal	USA	N/A	Obstructive airways disease (asthma)	21	AM3 (eResearch Technology)	FEV_1_
**Khan 2022 [41]**	Longitudinal	UK	70	Interstitial lung disease (interstitial lung disease)	82	Spirobank Smart (MIR)	FEV_1_
**Lindgren 1997 [42]**	Longitudinal	USA	48	Transplant (lung transplant)	77	PFM-H100 (Telemedical Inc.)	FEV_1_, FVC
**Marcoux 2019 [43]**	Longitudinal	Canada	73	Interstitial lung disease (idiopathic pulmonary fibrosis)	20	Spirometer (PMD Healthcare)	FVC
**Moor 2018 [44]**	Longitudinal	The Netherlands	71	Interstitial lung disease (idiopathic pulmonary fibrosis)	10	Spirobank Smart (MIR)	FEV_1_, FVC
**Moor 2019 [45]**	Longitudinal	The Netherlands	53^#^	Interstitial lung disease (pulmonary sarcoidosis)	10	Spirobank Smart (MIR)	FEV_1_, FVC
**Moor 2020a [46]**	Longitudinal	The Netherlands	70^#^	Interstitial lung disease (idiopathic pulmonary fibrosis)	46	Spirobank Smart (MIR)	FVC
**Moor 2020b [47]**	Longitudinal	The Netherlands	68^#^	Interstitial lung disease (interstitial lung disease)	50	Spirobank Smart (MIR)	FVC
**Moor 2021 [48]**	Longitudinal	Netherlands	60	Interstitial lung disease (interstitial lung disease)	10	Spirobank Smart (MIR)	FVC
**Morlion 2002 [49]**	Longitudinal	Belgium	33	Transplant (lung transplant)	22	Microloop II (MicroMedical)	FEV_1_, FEF_25–75%_
**Mortimer 2003^¶^ [50]**	Longitudinal	USA	9	Obstructive airways disease (asthma)	92	EasyOne (NDD Medical)	FEV_1_, FVC, FEF_25–75%_, PEF
**Odisho 2021 [51]**	Longitudinal	USA	N/A	Transplant (lung transplant)	311	Spirometer (Not reported)	FEV_1_
**Paynter 2021 [52]**	Longitudinal	USA	27	Suppurative lung disease (CF)	135	AM2+ (ERT Inc.)	FEV_1_
**Rodriguez-Roisin 2016 [53]**	Longitudinal	Global	64	Obstructive airways disease (COPD)	2488	EasyOne (NDD Medical)	FEV_1_
**Russell 2016 [54]**	Longitudinal	UK	67	Interstitial lung disease (idiopathic pulmonary fibrosis)	50	Microspirometer (CareFusion)	FEV_1_, FVC
**Shakkottai 2018 [55]**	Longitudinal	USA	16	Suppurative lung disease (CF)	39	Spiro PD (PMD Healthcare)	FEV_1_
**Sheshadri 2020 [56]**	Longitudinal	USA	55^#^	Transplant (haematopoietic cell transplantation)	82	GoSpiro (Monitored Therapeutics)	FEV_1_, FVC
**Turner 2021 [57]**	Longitudinal	USA	59^#^	Transplant (haematopoietic cell transplantation)	46	GoSpiro (Monitored Therapeutics)	FEV_1_, FVC
**Veit 2020 [58]**	Longitudinal	Germany	63	Interstitial lung disease (interstitial lung disease)	47	mySpirosense (PARI)	FVC
**Wijbenga 2020 [59]**	Longitudinal	The Netherlands	67	Transplant (lung transplant)	10	Spirobank Smart (MIR)	FEV_1_, FVC

Disease was classed as the predominant condition in the study population. CF: cystic fibrosis; FEV_1_: forced expiratory volume in 1 s; FVC: forced vital capacity; FEF_25–75%_: forced expiratory flow at 25–75% of the FVC; PEF: peak expiratory flow; COPD: chronic obstructive pulmonary disease. ^#^: median age reported; ^¶^: studies in children (Kerwin *et al.* [40] reports outcomes of a subanalysis of 12–17-year-olds).

### Risk of bias

For the QUADAS risk of bias assessment (supplementary material), five studies were assessed as having an overall low risk of bias across all domains. 23 studies were assessed as at risk of bias due to one or more domains being at high or unclear risk, as per reasons described within the Methods section.

### FEV_1_

**FIGURE 2:**
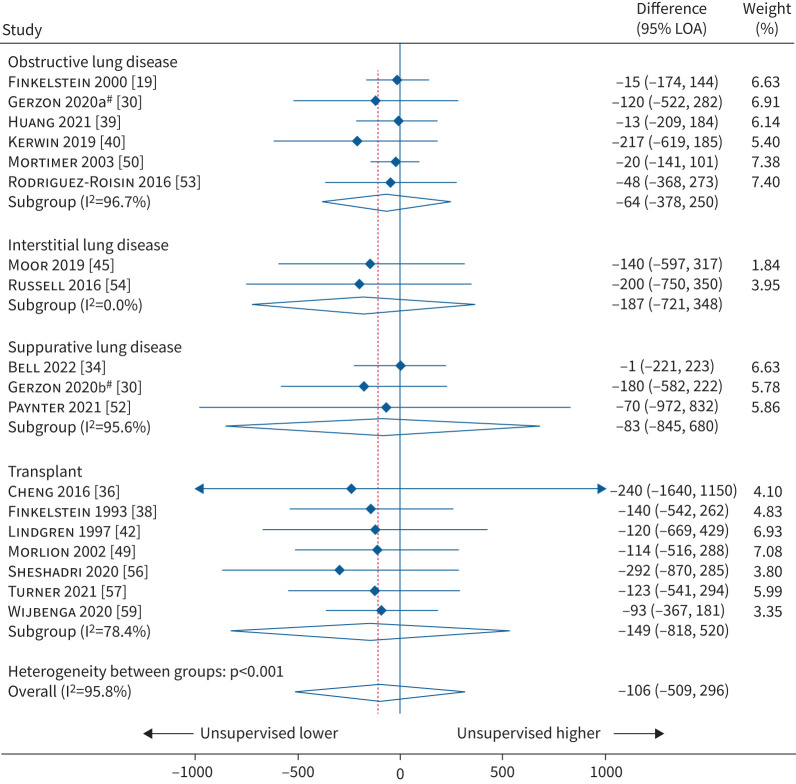
Meta-analysis of Bland–Altman for forced expiratory volume in 1 s (in mL) according to type of disease. LoA: limits of agreement. ^#^: Gerzon *et al.* [30] reported values for asthma (2020a) and cystic fibrosis patients (2020b) separately and these were treated as two separate studies.

For the primary outcome of FEV_1_, 17 studies (4517 patients; 9855 data comparison points) reported Bland–Altman data for FEV_1_. Pooled analysis showed the overall mean difference for FEV_1_ between unsupervised and supervised spirometry was −106 mL lower when unsupervised (LoA= −509 mL, 296 mL; I^2^=95.8%; p≤0.001; figure 2). Six studies included patients with obstructive lung disease with a mean difference of −64 mL (LoA= −378 mL, 250 mL; I^2^=96.7%). Two studies included patients with interstitial lung disease with a mean difference of −187 mL (LoA= −721 mL, 348 mL; I^2^=0.0%). Three studies included patients with suppurative lung disease with a mean difference of −83 mL (LoA= −845 mL, 680 mL; I^2^=95.6%). Seven studies included transplant patients with a mean difference of −149 mL (LoA= −818 mL, 520 mL; I^2^=78.4%).

A subgroup analysis of four studies that had a low risk of bias found a mean difference of −103 mL (LoA= −444 mL, 238 mL; I^2^=96.2%; table 2) between unsupervised and supervised measurements. The 14 studies that were at risk of bias had a mean difference of −115 mL (LoA= −705 mL, 475 mL; I^2^=95.9%). Subgroup analysis for studies that compared unsupervised and supervised measurements within the same day and studies with adults and children are shown in table 2. The forest plots for these analyses are in the supplementary material. There was noticeable asymmetry with funnel plot estimates for FEV_1_ (supplementary material). In addition, 12 studies reported correlation values for FEV_1_ with an overall median of 0.949 (IQR 0.855, 0.982; supplementary material).

**TABLE 2:** Summary of findings and subgroup analyses

**Outcomes and subgroups**	**Participants (n)**	**Studies (n)**	**Mean difference (unsupervised− supervised)** ^#^	**LoA**	**95% CI**	**I^2^ (%)**	**Anticipated impact (%)** ^¶^
**FEV_1_ overall**	4517	17	**−**106 mL	**−**509, 296	−129, −84	95.8	38.9
Low risk of bias	2679	4	**−**103 mL	**−**444, 238	−160, −46	96.2	38.6
At risk of bias	1178	14	**−**115 mL	**−**705, 475	−154, −75	95.9	39.9
Same day measurements	235	5	**−**65 mL	**−**421, 292	−129, −1	95.4	35
Non-same day measurements	3520	11	**−**144 mL	**−**561, 272	−184, −105	95.3	43.8
Adults	3627	14	**−**103 mL	**−**517, 311	−134, −73	93.6	38.6
Children	230	4	**−**132 mL	**−**410, 146	−222, −42	98.2	42.1
**FVC overall**	1307	17	**−**184 mL	−1028, 660	**−**253, **−**114	96	66.9
Low risk of bias	238	4	**−**118 mL	**−**886, 650	**−**201, **−**35	88.5	65.2
At risk of bias	1069	13	**−**207 mL	**−**1098, 684	**−**300, **−**114	96.6	67.7
Same day measurements	106	2	**−**14 mL	**−**287, 260	**−**42, 15	0	63.9
Non-same day measurements	1099	13	**−**228 mL	**−**1239, 783	**−**285, **−**171	85.3	68.4
Adults	1215	16	**−**198 mL	**−**1162, 766	**−**262, **−**134	92.5	67.7
**FEF_25–75%_**	146/899	3	**−**19 mL·s^−1^	**−**497, 458	**−**329, 291	99.7	41.3
**PEF**	124/400	2	**−**92 mL·s^−1^	**−**498, 314	**−**110, **−**74	0	38.1

The standard deviation was imputed for five studies so mean differences could be calculated after efforts were made to obtain data from authors [30, 37, 38, 40, 49]. LoA: limits of agreement; FEV_1_: forced expiratory volume in 1 s; FVC: forced vital capacity; FEF_25–75%_: forced expiratory flow at 25–75% of the FVC; PEF: peak expiratory flow. ^#^: a negative mean difference indicates unsupervised spirometry is lower. ^¶^: percentage of results that would probably have a difference >200 mL.

### FVC

**FIGURE 3:**
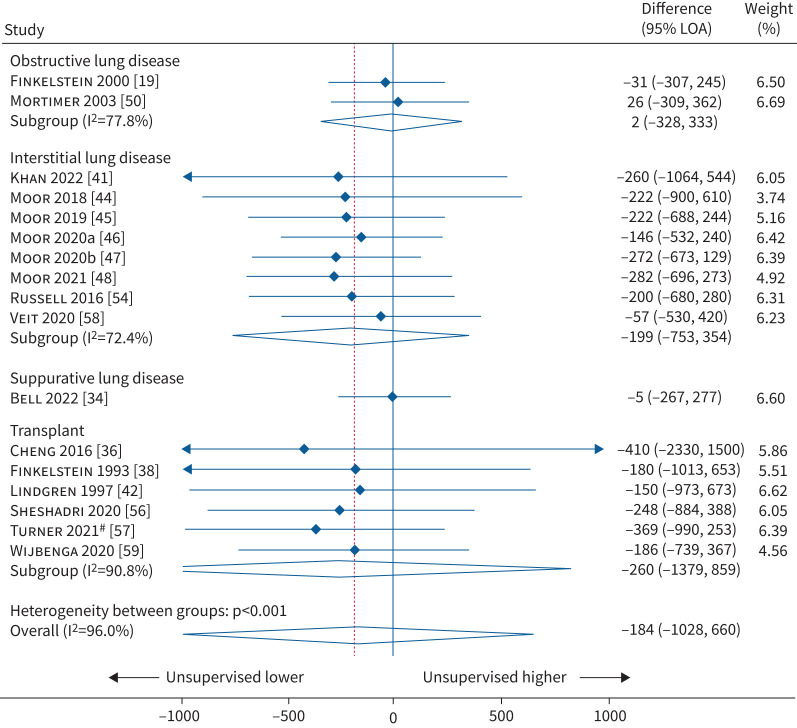
Meta-analysis of Bland–Altman for forced vital capacity (FVC) (in mL) according to type of disease. LoA: limits of agreement. ^#^: Turner *et al.* [57] used forced expiratory volume in 6 s (FEV_6_) as a surrogate for FVC.

17 studies (n=1307 patients; 1926 data comparison points) reported Bland–Altman data for FVC, with pooled analysis showing the overall mean difference between unsupervised and supervised spirometry was −184 mL lower when unsupervised (LoA= −1028 mL, 660 mL; I^2^=96%; p<0.001; figure 3). Two studies included patients with obstructive lung disease with a mean difference of 2 mL (LoA= −328 mL, 333 mL; I^2^=77.8%). Eight studies included patients with interstitial lung disease with a mean difference of −199 mL (LoA= −753 mL, 354 mL; I^2^=72.4%). One study included patients with suppurative lung disease with a mean difference of −5 mL (LoA= −267 mL, 277 mL). Six studies included transplant patients with a mean difference of −260 mL (LoA= −1379 mL, 859 mL; I^2^=90.8%).

A subgroup analysis of four studies that had a low risk of bias found a mean difference of −118 mL (LoA= −886 mL, 650 mL; I^2^=88.5%; table 2) between unsupervised and supervised measurements. The 13 studies that were at risk of bias had a mean difference of −207 mL (LoA= −1098 mL, 684 mL; I^2^=96.6%). Subgroup analysis for studies that compared unsupervised and supervised measurements within the same day and studies with adults and children are shown in table 2. The forest plots for these analyses are in the supplementary material. There was noticeable asymmetry with funnel plot estimates (supplementary material). In addition, 14 studies reported correlation values for FVC with an overall median of 0.967 (IQR 0.940, 0.976; supplementary material).

### FEF_25–75%_ and PEF

Three studies reported FEF_25–75%_ (146 patients; 899 data comparison points) with an overall mean difference of −19 mL·s^−1^ when unsupervised (LoA= −497 mL·s^−1^, 458 mL·s^−1^; I^2^=99.7%). Two studies reported PEF (124 patients; 400 data comparison points) with an overall mean difference of −92 mL·s^−1^ (LoA= −498 mL·s^−1^, 314 mL·s^−1^; I^2^=0.0%). The forest plots for these analyses are in the supplementary material.

### Secondary outcomes

The secondary outcomes of adherence, patient satisfaction/acceptability, technical issues, quality of spirometry data, adverse events and costs are summarised in the supplementary material.

### GRADE

For the primary outcome, the certainty of the evidence was deemed very low for all four measures (supplementary material).

## Discussion

Despite the rationale for why unsupervised spirometry might be useful for monitoring respiratory patients with respiratory conditions [17, 18], we found that on average unsupervised spirometry measurements were lower (FEV_1_ and FVC) than supervised measurements, with wide variability. This is contrary to the interpretations in some studies that have stated that unsupervised spirometry values are acceptable. However, we found that the large variation has been obscured in many cases because of a focus on the mean differences with confidence intervals and correlations. We assert this is not the most appropriate analysis to compare agreement by two methods of measurement as demonstrated by Bland and Altman [60], who highlighted the misconception that correlation is evidence for agreement and suggested mean differences and LoA are needed [61], as was used in this review. We found a substantial percentage of results would be expected to have a difference >200 mL (table 2). Two clinical examples highlight important implications from the study findings (table 3).

**TABLE 3:** Real-world clinical examples that could result from the findings of this review

**Clinical scenario**	**Implications**
A patient completes baseline FEV_1_ spirometry supervised in clinic and subsequently completes spirometry unsupervised at home and obtains the same results.	The results from this systematic review indicate that, based on the CI and LoA for FEV_1_, a clinician cannot be confident that the results were truly the same. Despite there being no apparent change in their underlying lung function, there is the chance of a mean underestimation of 106 mL with home spirometry and a 39% chance that the difference would be >200 mL from the supervised clinic FEV_1_ measurement.
A patient who feels unwell at home records a remote FEV_1_ spirometry measurement that is >10% lower than a previous supervised clinic measurement, a change frequently used as an indicator for bronchiectasis exacerbations.	As described in the example above, this difference could be due to chance or could indicate a true decline in lung function. Crucially, if the home and clinic results appeared the same then important changes in the patient's health could be missed, due to false reassurance to the patient and clinician.

FEV_1_: forced expiratory volume in 1 s; LoA: limit of agreement; CI: confidence interval.

For studies with a low risk of bias (table 2 and supplementary material), there were acceptable mean differences between the two methods, based on ATS/ERS repeatability criteria [1]. This suggests that unsupervised lung function might be useful as an outcome measure for the follow-up of groups of patients in large research studies because it is likely that the average results represent the population mean. However, the large variation and high heterogeneity reflect diverse patient demographics and different spirometry methods used across the studies.

Recently, the landscape of spirometry has changed, with new portable devices available for use by patients. Although most studies in this review were published from 2016 onwards, it is likely that future studies will experience a positive learning curve, meaning a greater number of patients will have prior experience with portable spirometers and might produce more accurate results unsupervised [41, 58]. This could also apply to the training of clinicians [53, 58]. The ARTP recommend that clinicians complete a practical examination and perform at least 50 spirometry tests on patients per year to remain competent in spirometry, and to evidence quality and consistency, highlighting the technicality of the procedures [12]. We advise relevant organisations to consider the increase in unsupervised spirometry and provide guidance on optimisation and use of home measurements. Furthermore, any new portable spirometers must be thoroughly validated, because a recent review found that only three of 10 devices from various manufacturers were technically acceptable [62].

Most studies that explored adherence reported good compliance to home spirometry. Patient satisfaction was also generally positive towards the portable spirometers and technical issues appeared to be low. However, a wide variety of methods were used to determine these outcomes. No costs were reported but these can be substantial, especially when paired with new airway clearance devices in some diseases [63].

Regarding strengths, this is the first review to comprehensively search for studies to estimate the difference between unsupervised and supervised spirometry. The large number of patients, conditions and comparisons provide strength to the meta-analysis. In the screening stage of the review, we identified several studies that reported feasibility with portable spirometers when used by patients in the presence of clinicians, which is likely to influence the measurements, making it indistinguishable from a true unsupervised measurement. On this basis, such studies were not eligible for this review, and we only included studies to represent what would happen in real-world situations.

A limitation of the findings is the statistical heterogeneity in the pooled analysis and very low certainty of the evidence. Although there was significant heterogeneity in the magnitude of differences across all outcomes, unsupervised measurements were consistently lower than clinic measurements in almost all studies. We were unable to create a combined Bland–Altman plot to assess patterns of variability because individual patient values were not available. Furthermore, this was a cross-sectional analysis and did not explore successive measurements. Most studies were deemed at risk due to unclear methods. Some studies did not fully report metrics around the Bland–Altman analysis. We recommend that future studies report this fully [64]. Future reviews should explore the FEV_1_/FVC ratio and longitudinal analysis of data across time. As outlined in the new ERS/ATS guidelines [65], it is important we understand the reproducibility of spirometry measurements (unsupervised and supervised) and what indicates a clinically meaningful change over time. The majority of studies did not explicitly report if supervised measurements were performed under the instruction of a clinician or other trained professional. In addition, the majority of studies did not report if follow-up training on the quality of spirometry technique was performed with patients after the baseline visit to improve the quality of unsupervised measurements. The studies did not comprehensively describe how quality assessment of the spirometry was performed. Future studies should aim to provide more detail on aspects of patient training and quality assessment for both supervised and unsupervised spirometry in their methodology.

In conclusion, unsupervised home spirometry underestimates lung function measurements compared to supervised spirometry. We suggest caution and proper training if used owing to the possibility of underestimation and large variation in the differences between unsupervised and supervised measurements. Unsupervised home spirometry should not be used for diagnostic purposes; however, the results do suggest that unsupervised measurements may be suitable for outcome collection within large clinical research studies. The focus here is likely to be on the mean difference in lung function between the study groups and a large sample size could overcome the added measurement variation, and so represent the population mean. Any future research should use technically validated devices in a comprehensively trained population across multiple timepoints to fully understand the value of unsupervised home spirometry.

**Points for clinical practice**

It is crucial to know whether assessments, such as FEV_1_ and FVC, taken by unsupervised patients are consistent with measurements taken in a clinical setting with a trained professional. Based on this review, we urge caution when using unsupervised spirometry for individual patients and suggest clinicians consider the potential for differences because measurements are not interchangeable and can result in underestimation.

## Supplementary material

10.1183/16000617.0248-2022.Supp1 **Please note:** supplementary material is not edited by the Editorial Office, and is uploaded as it has been supplied by the author.
Supplementary material ERR-0248-2022.SUPPLEMENT
