# Supplementary material for: Unsupervised home spirometry versus supervised clinic spirometry for respiratory disease: a systematic methodology review and meta-analysis
Source: Eur Respir Rev. 2023 Sep 6;32(169):220248. doi: 10.1183/16000617.0248-2022 (PMC10481332; doi:10.1183/16000617.0248-2022)
Supplement: Supplementary file 1 [file ERR-0248-2022.SUPPLEMENT.pdf]

Supplementary Material

Table of Contents

Unsupervised definition: 2
Search strategies: 2
QUADAS-2 ROB form: 7
Risk of Bias visualization 9
FEV_1 Subgroup analysis (study quality/risk of bias) 10
FEV_1 Subgroup Analysis (Same day comparison) 11
FEV_1 Subgroup Analysis (Children and Adults) 12
FEV_1 Funnel Plot Estimate 13
FVC Subgroup analysis of (study quality/risk of bias) 13
FVC Subgroup Analysis (Same day comparison) 14
FVC Subgroup Analysis (Adults) 15
FVC Funnel Plot Estimate 15
$FEF_{25–75}$ forest plot 16
PEF forest plot 16
Secondary outcomes (adherence, patient satisfaction/acceptability, technical issues, quality of spirometry, adverse events and cost). 17
Adherence 19
Patient satisfaction/acceptability: 20
Technical issues: 21
Meta-analyses of forest plots using confidence intervals (Cis) 22
    FEV_1 22
    FVC 23
    FEF 23
    PEF 24
FEV_1 Correlation 24
FVC Correlation 25
FEF Correlation 25
PEF Correlation 25
GRADE certainty of evidence table 26
Variations between review and protocol 27

Unsupervised definition:

For this review, 'unsupervised' was defined as the participants completing spirometry in the absence of a clinician or other professional support and could be at any location. 'Supervised' was defined as the presence of a clinician or other trained professional along with the participant when they completed spirometry, again at any location.

Search strategies:

MEDLINE search (15th July 2021):

Method (spirometry):

1. exp Spirometry/
2. Spirometry.mp.
3. Lung function.mp.
4. Lung assessment.mp.
5. Pulmonary function.mp.
6. Pulmonary test.mp.
7. Pulmonary measurement.mp.
8. Pulmonary assessment.mp.
9. Exp Respiratory function tests/
10. Respiratory function.mp.
11. Respiratory test.mp.
12. Respiratory measurement.mp.
13. Respiratory assessment.mp.
14. Airways function.mp.
15. Forced expiratory volume.mp. or exp Forced Expiratory Volume/
16. Forced vital capacity.mp. or exp Vital Capacity/
17. Forced Expiratory Flow.mp.
18. Peak Expiratory Flow Rate/ or Peak expiratory flow.mp.
19. Peak flow measurement.mp.
20. Or/1-19

Method (remote):

21. Remote Consultation.mp. or exp Remote Consultation/
22. Unsupervised.mp.
23. Unassisted.mp.
24. Patient-led.mp.
25. telemedicine.mp. or exp Telemedicine/
26. Telemonitoring.mp.
27. Tele-monitoring.mp.
28. Virtual visit.mp.
29. Virtual.mp
30. Or/21-29

Type of study:

31. randomized controlled trial.mp. or exp Randomized Controlled Trial/
32. randomised controlled trial.mp.
33. RCT.mp.
34. controlled clinical trial.mp. or exp Controlled Clinical Trial/
35. controlled study.mp.
36. clinical trial.mp. or exp Clinical Trial/
37. clinical study.mp. or exp Clinical Study/
38. comparative study.mp. or exp Comparative Study/
39. exp evaluation studies/
40. prospective study.mp. or exp Prospective Studies/
41. Cross-Sectional Studies/
42. Longitudinal Studies/
43. exp Observational Study/
44. exp Cohort Studies/
45. non-randomised.mp.
46. or/31-45

47. 20 and 30 and 46
48. limit 46 to (english language and humans)

Number of search results

MEDLINE: 441

Number of duplicates removed: 228

Total number to screen in Covidence: 213

EMBASE Search (15th July 2021):

Method (spirometry):

1. Spirometry.mp. or exp Spirometry/
2. lung function.mp. or exp lung function/
3. Lung assessment.mp.
4. Pulmonary function.mp.
5. Pulmonary test.mp.
6. Pulmonary measurement.mp.
7. Pulmonary assessment.mp.
8. respiratory function test.mp. or exp lung function test/
9. respiratory function.mp. or respiratory function/
10. Respiratory test.mp.
11. Respiratory measurement.mp.
12. Respiratory assessment.mp.
13. Airways function.mp.
14. Forced expiratory volume.mp. or exp Forced Expiratory Volume/
15. Forced vital capacity.mp. or exp Forced Vital Capacity/
16. forced expiratory flow.mp. or exp forced expiratory flow/
17. peak expiratory flow.mp. or exp peak expiratory flow

18. Peak flow measurement.mp.
19. Or/1-18

Method (remote):

20. remote consultation.mp. or exp teleconsultation/
21. Unsupervised.mp.
22. Unassisted.mp.
23. Patient-led.mp.
24. telemedicine.mp. or exp telemedicine/
25. telemonitoring.mp. or exp telemonitoring/
26. Tele-monitoring.mp.
27. exp home visit/ or home respiratory care/ or virtual visit.mp
28. remote visit.mp
29. Or/20-28

Type of study:

30. randomised controlled trial.mp. or exp Randomized Controlled Trial/
31. randomized controlled trial.mp.
32. RCT.mp.
33. controlled clinical trial.mp. or exp Controlled Clinical Trial/
34. controlled study.mp. or exp controlled study/
35. clinical trial.mp. or exp Clinical Trial/
36. clinical study.mp. or exp Clinical Study/
37. comparative study.mp. or exp Comparative Study/
38. exp evaluation studies/
39. Longitudinal Study/
40. exp Observational Study/
41. cohort study.mp.
42. non-randomised.mp.
43. or/30-42

44. 19 and 29 and 43
45. limit 44 to (english language and humans)

Number of search results

EMBASE: 967
Number of duplicates removed: 81
Total number to screen in Covidence: 886

Cochrane Library Search (15th July 2021):

Method (spirometry):

1. MeSH: [Spirometry] explode all trees
2. MeSH: [Respiratory Function Test] explode all trees
3. “Lung assessment”
4. "pulmonary assessment"
5. “Airways function”

6. MeSH: [Forced Expiratory Volume] explode all trees
7. MeSH: [Forced Vital Capacity] explode all trees
8. MeSH: [Forced expiratory flow rates] explode all trees
9. “forced expiratory flow”
10. MeSH: [peak expiratory flow rate] explode all trees
11. “peak expiratory flow”
12. #1 or #2 or #3 or #4 or #5 or #6 or #7 or #8 or #9 or #10 or #11

<u>Method (remote):</u>

13. MeSH: [remote consultation] explode all trees
14. Unsupervised
15. Unassisted
16. Patient-led
17. MeSH: [telemedicine] explode all trees
18. telemonitoring
19. Tele-monitoring
20. MeSH: [house calls] explode all trees
21. “virtual visit”
22. MeSH: [telerehabilitation]
23. “remote visit”
24. #13 or #14 or #15 or #16 or #17 or #18 or #19 or #20 or #21 or #22 or #23

<u>Type of study:</u>

25. MeSH: [Randomized Controlled Trials as Topic] explode all trees
26. “randomized controlled trial”
27. MeSH: [Controlled Clinical Trial as Topic] explode all trees
28. “controlled study”
29. “comparative study”
30. MeSH: [Proof of Concept Study] explode all trees
31. MeSH: [Clinical trials as topic] explode all trees
32. MeSH: [Clinical studies as topic] explode all trees
33. MeSH: [comparative study] explode all trees
34. MeSH: [Longitudinal Studies] explode all trees
35. MeSH: [Observational Studies] explode all trees
36. MeSH: [cohort studies] explode all trees
37. #25 or #26 or #27 or #28 or #29 or #30 or #31 or #32 or #33 or #34 or #35 or #36

38. #12 and #24 and #37
39. limit 38 to (Cochrane Protocols and Trials)

Number of search results

Cochrane Central Library: 148
Number of duplicates removed: 4
Total number to screen in Covidence: 144

Open Access Theses and Dissertations search (16th July 2021):
Search limiters: English only
Search any field with:

1. Home spirometry = 21
2. Remote spirometry = 4 (1 excluded as non-English)
3. Home "lung function" = 30
4. Remote "lung function" = 2

Total number of search results: 56
Number of duplicates removed: 11
Total number outside of Covidence: 45

Reference lists of systematic reviews (23rd August 2021):
Total number references screened: 316
Number of duplicates removed: 4
Total number to screen in Covidence: 22

Reference lists of included studies (22nd November 2021):
Total number references screened: 610
Number of duplicates removed: n/a
Total number to screen in Covidence: 2

Additional papers added from other sources (23rd August 2021):
Total number to screen in Covidence: 1

Additional PubMed search to find key terms "portable" and "handheld" (23rd August 2021):

((((portable [Title]) AND (spirometry[Title])) OR ((handheld[Title]) AND (spirometry[Title]))) OR ((handheld[Title]) AND (spirometer[Title]))) OR ((portable[Title]) AND (spirometer[Title]))

Total number of search results: 64
Number of duplicates removed: 3
Total number to screen in Covidence: 61

QUADAS-2 ROB form:

Title of record:

Record number on Covidence: #
Name of assessor:
Date of assessment:

QUADAS-2 tool: Risk of bias and applicability judgments

**Domain 1: Patient selection**	
**A. Risk of bias**	
**Describe methods of patient selection:**	
• **Was a consecutive or random sample of patients enrolled?**	Yes/No/Unclear
• **Was a case-control design avoided?**	Yes/No/Unclear
**Could the selection of patients have introduced bias?**	RISK: LOW/HIGH/UNCLEAR

**Domain 2: Index test (unsupervised spirometry)**	
**A. Risk of bias**	
**Describe the index test and how it was conducted and interpreted:**	
**Could the conduct or interpretation of the index test have introduced bias? If HIGH, please explain:**	RISK: LOW/HIGH/UNCLEAR

**Domain 3: Reference standard (supervised spirometry)**	
**A. Risk of bias**	
**Describe the reference standard and how it was conducted and interpreted:**	
• **Is the reference standard likely to correctly classify the target condition of the study?**	Yes/No/Unclear
**Could the reference standard, its conduct, or its interpretation have introduced bias? If HIGH, please explain:**	RISK: LOW/HIGH/UNCLEAR
**B. Concerns regarding applicability**	
**Is there concern that the reference test, its conduct, or interpretation differ from a reference standard (e.g. ATS/ERS standards)? If HIGH, please explain:**	CONCERN: LOW/HIGH/UNCLEAR

Domain 4: Patient flow and timing of tests

A. Risk of bias

Describe any patients who did not receive the index test(s) and/or reference standard or who were excluded (number of withdrawals):

Describe the time interval between index test(s) and reference standard in the primary analysis:

Describe any interventions between index test(s) and reference standard (i.e. different package of care

• **Was there an appropriate interval between index test(s) and reference standard?**	Yes/No/Unclear
• **Did all patients receive a reference standard?**	Yes/No/Unclear
• **Did patients receive the same reference standard (same type of spirometer)?**	Yes/No/Unclear
• **Were all patients included in the analysis?**	Yes/No/Unclear
**Could the patient flow and/or spirometry timing have introduced bias? If HIGH, please explain:**	RISK: LOW/HIGH/UNCLEAR

Risk of Bias visualization

for included studies assessed using the QUADAS-2 tool. If a study was judged “high” or “unclear” in 1 or more domains, then it was “at risk of bias”. D= Domain. Studies ordered alphabetically by first author.

STUDY	RISK OF BIAS					
	D1: PATIENT SELECTION	D2: INDEX TEST (unsupervised)	D3: REFERENCE STANDARD (supervised)	D3: APPLICABILITY CONCERNS	D4: FLOW AND TIMING	OVERALL RISK
Bell 2022	Low	High	Low	Low	Unclear	At risk of bias
Broos 2018	Unclear	Unclear	Unclear	Unclear	Unclear	At risk of bias
Cheng 2016	Low	Low	Low	Low	High	At risk of bias
Edmondson 2021	Unclear	Unclear	Unclear	Unclear	Unclear	At risk of bias
Finkelstein 1993	Unclear	Low	Low	Unclear	Unclear	At risk of bias
Finkelstein 2000	Low	Low	Low	Low	Low	Low
Gerzon 2020	Unclear	Low	Low	Low	Unclear	At risk of bias
Huang 2021	Unclear	Low	Low	Low	High	At risk of bias
Kerwin 2019	Low	Unclear	Low	Unclear	Unclear	At risk of bias
Khan 2022	Low	High	Unclear	Low	Unclear	At risk of bias
Lindgren 1997	Low	Low	Low	Low	Low	Low
Marcoux 2019	Unclear	Low	Low	Low	Unclear	At risk of bias
Moor 2018	Low	Low	Unclear	Unclear	Low	At risk of bias
Moor 2019	Low	Low	Unclear	Unclear	Unclear	At risk of bias
Moor 2020a	Low	Low	Low	Unclear	Low	At risk of bias
Moor 2020b	Low	Unclear	Unclear	Unclear	Unclear	At risk of bias
Moor 2021	Low	Low	Unclear	Unclear	High	At risk of bias
Morlion 2002	Low	Low	Unclear	Unclear	Low	At risk of bias
Mortimer 2003	Low	Low	Low	Low	Unclear	At risk of bias
Odisho 2021	Unclear	Unclear	Unclear	Unclear	High	At risk of bias
Paynter 2021	Low	Low	Low	Low	High	At risk of bias
Rodriguez-Roisin 2016	Low	Low	Low	Low	Low	Low
Russell 2016	Low	High	Low	Low	Low	At risk of bias
Shakkottai 2018	Unclear	Low	Unclear	Unclear	Unclear	At risk of bias
Sheshadri 2020	Low	Low	Low	Low	Low	Low
Turner 2021	Unclear	Low	Low	Low	Unclear	At risk of bias
Veit 2020	Low	Low	Low	Low	Low	Low
Wijbenga 2020	Low	Low	Unclear	Unclear	High	At risk of bias

Low Risk	Unclear Risk	High Risk

FEV$_1$ Subgroup analysis (study quality/risk of bias)

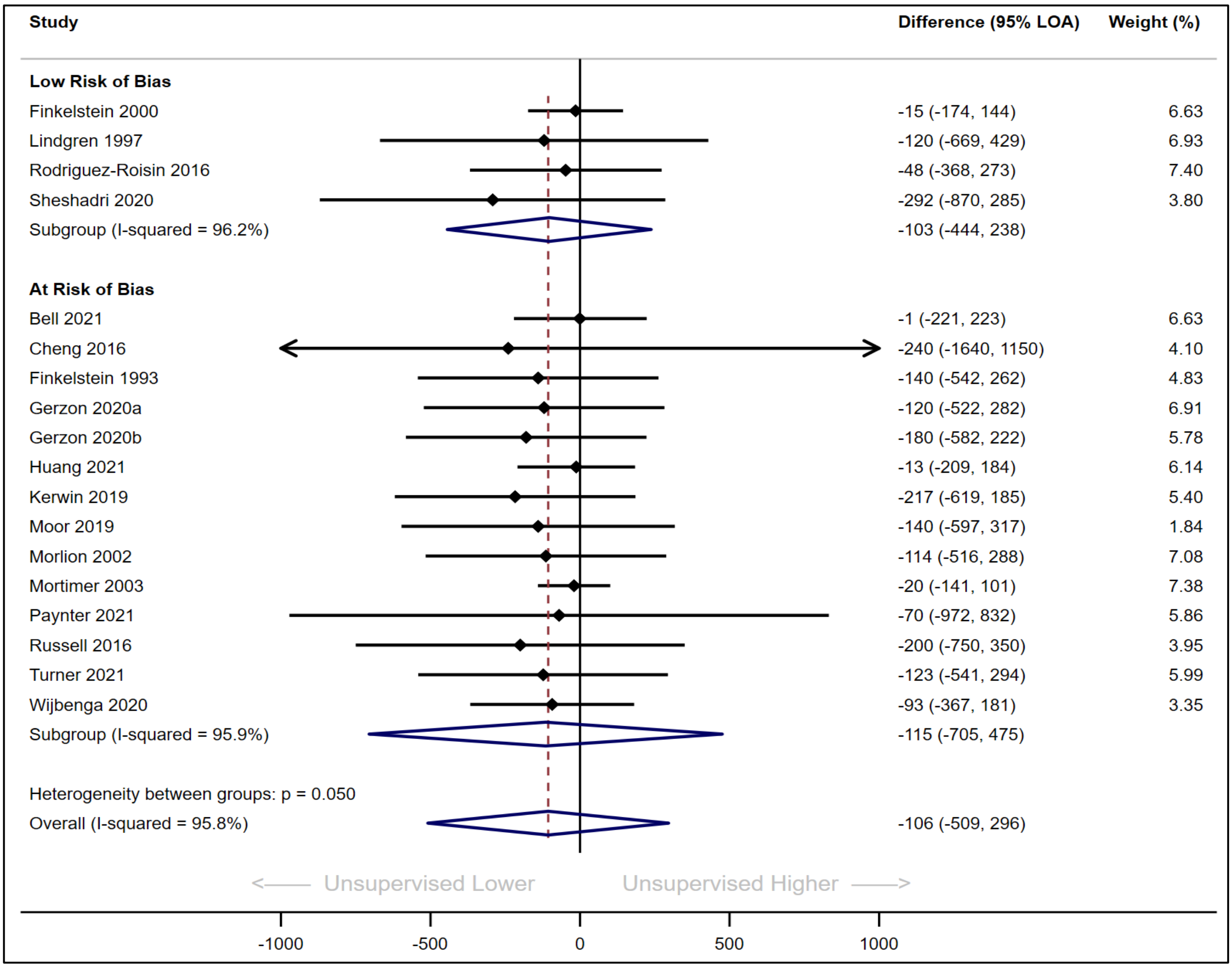

FEV$_1$ Subgroup Analysis (Same day comparison)

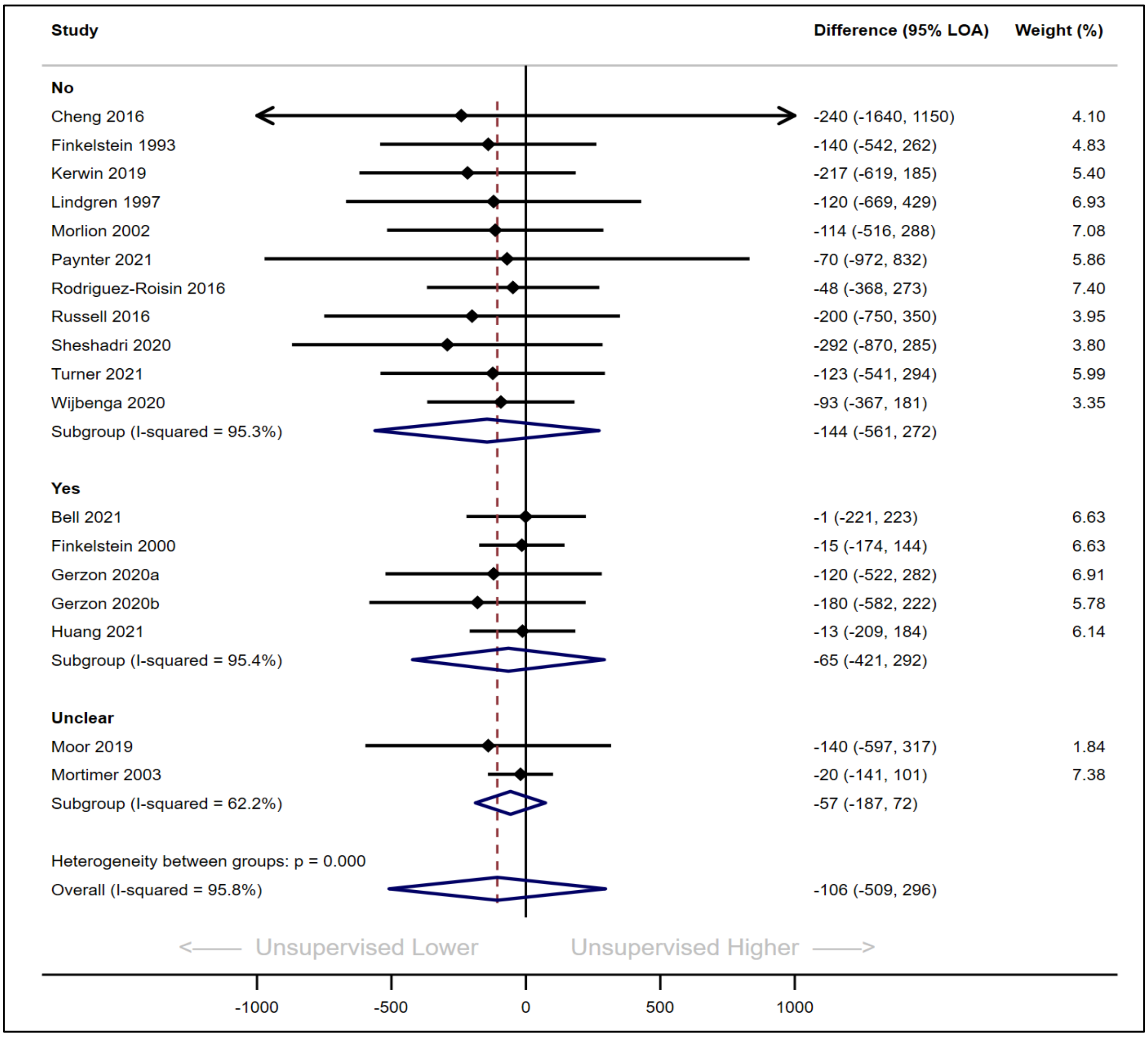

FEV_1 Subgroup Analysis (Children and Adults)

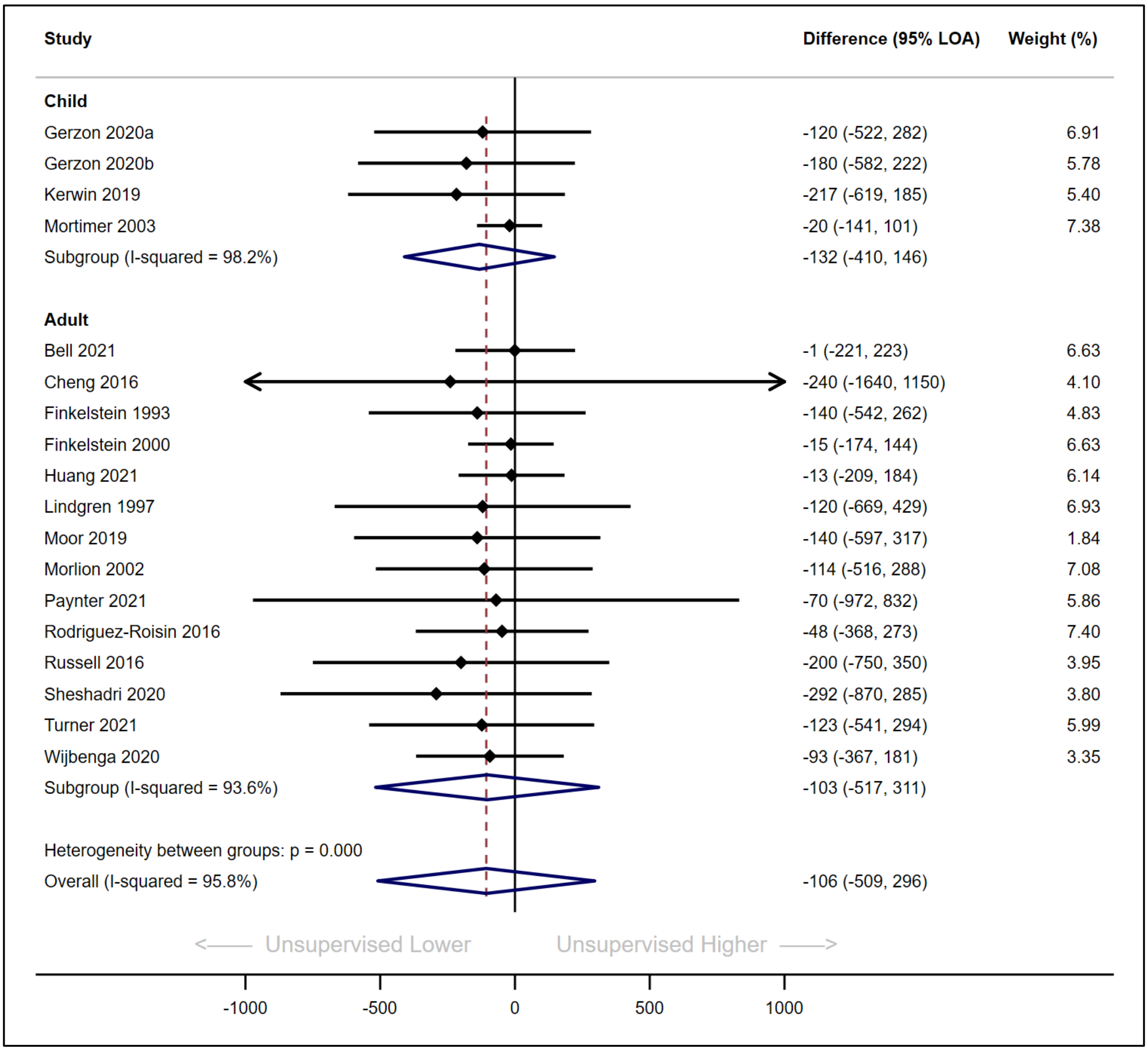

FEV_1 Funnel Plot Estimate

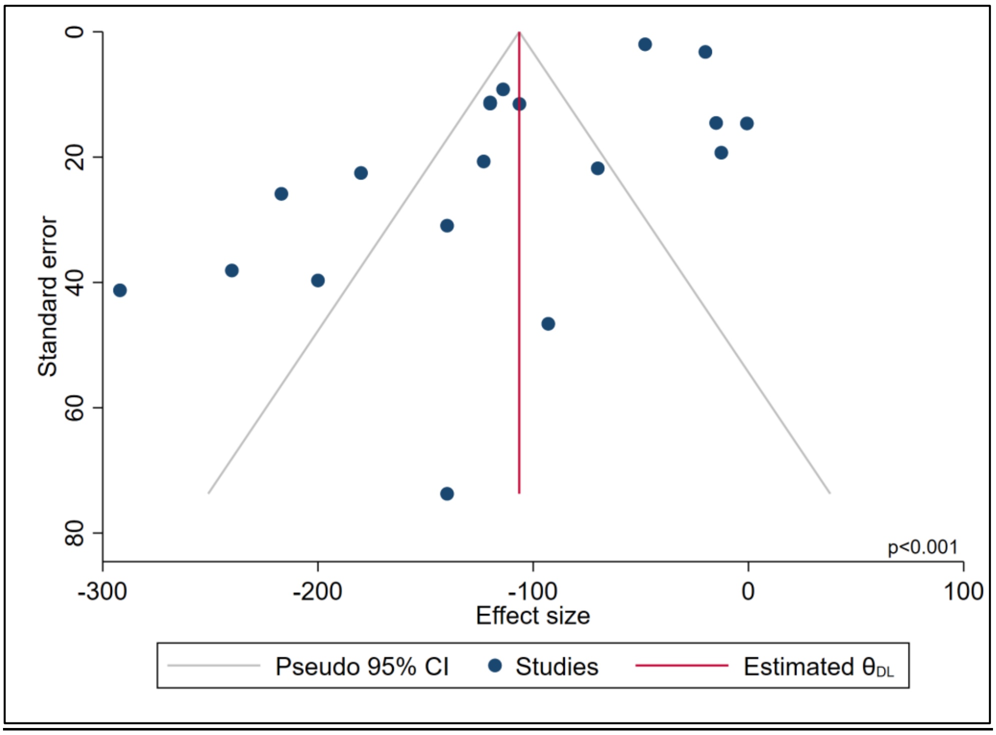

FVC Subgroup analysis of (study quality/risk of bias)

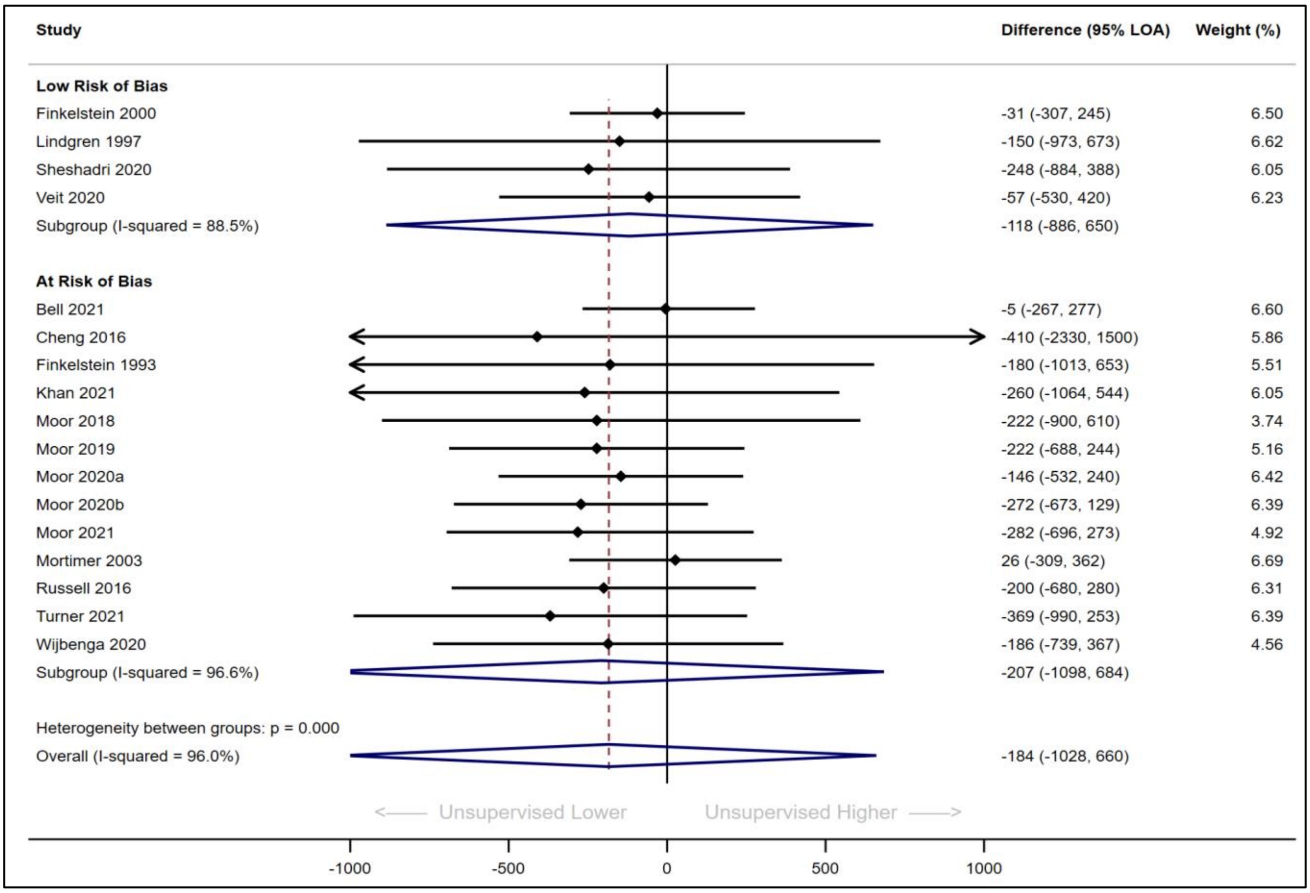

FVC Subgroup Analysis (Same day comparison)

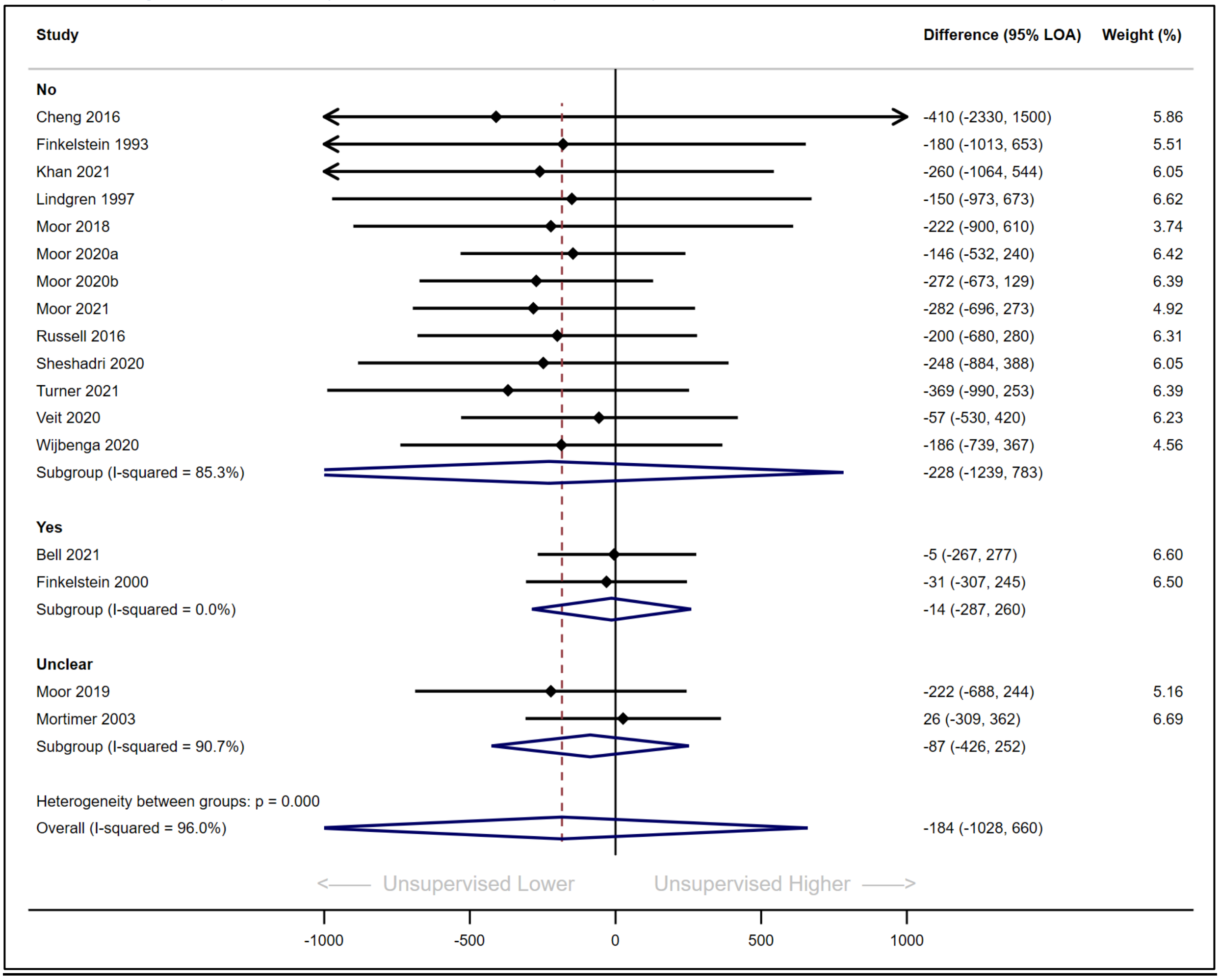

FVC Subgroup Analysis (Adults)

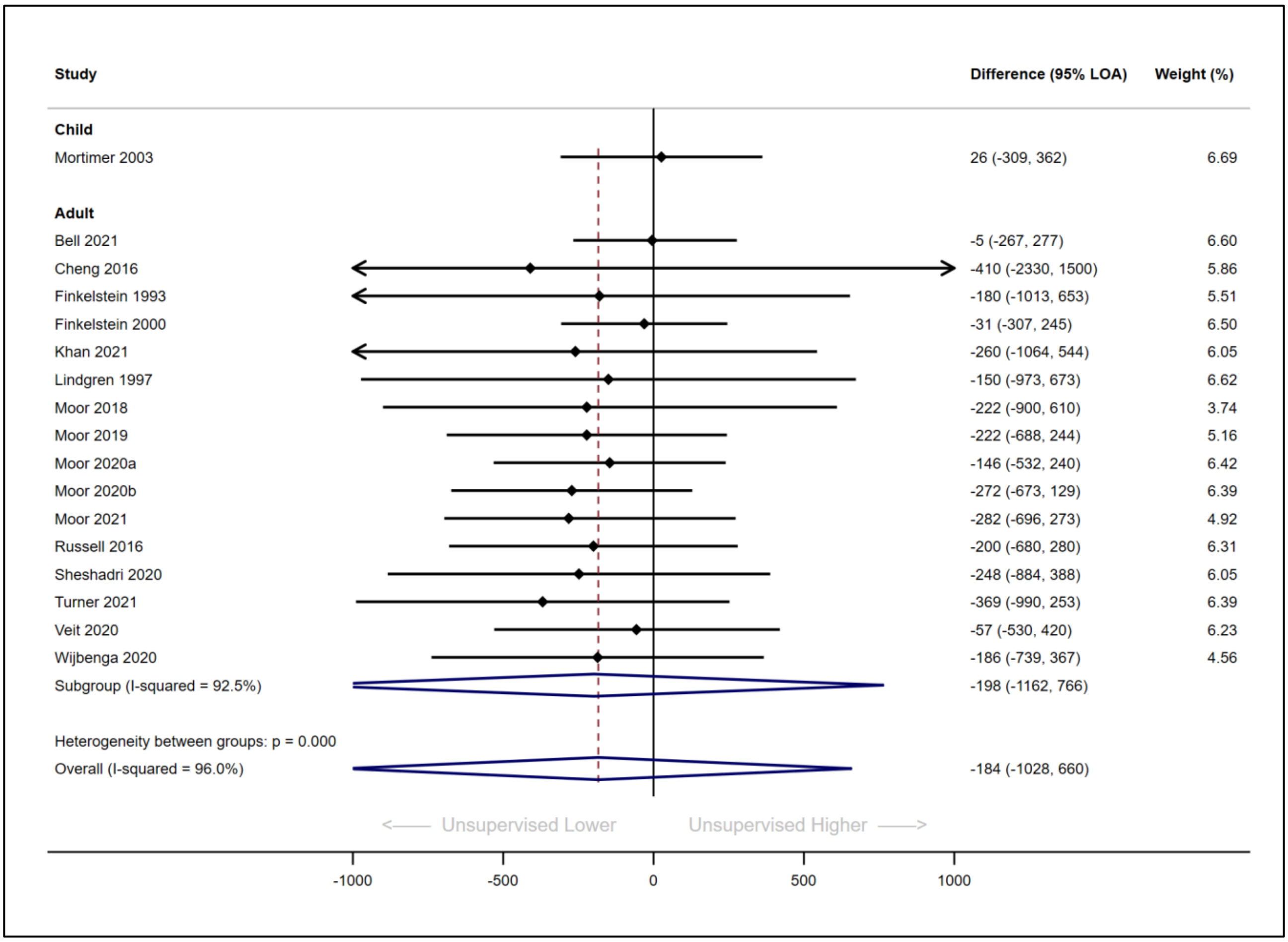

FVC Funnel Plot Estimate

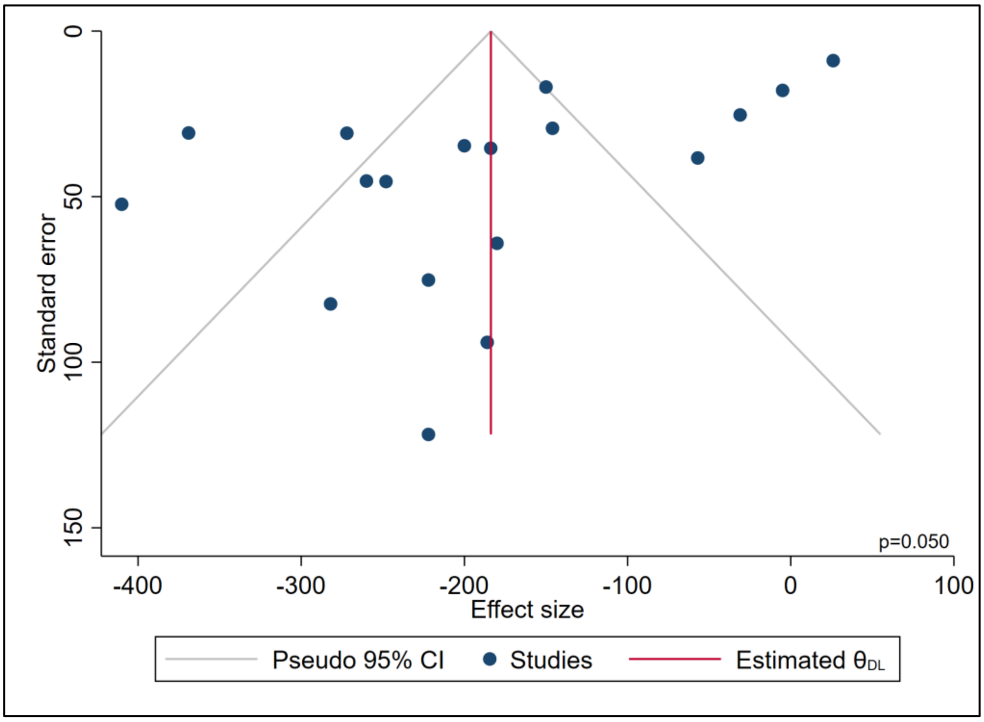

FEF$_{25–75}$ forest plot

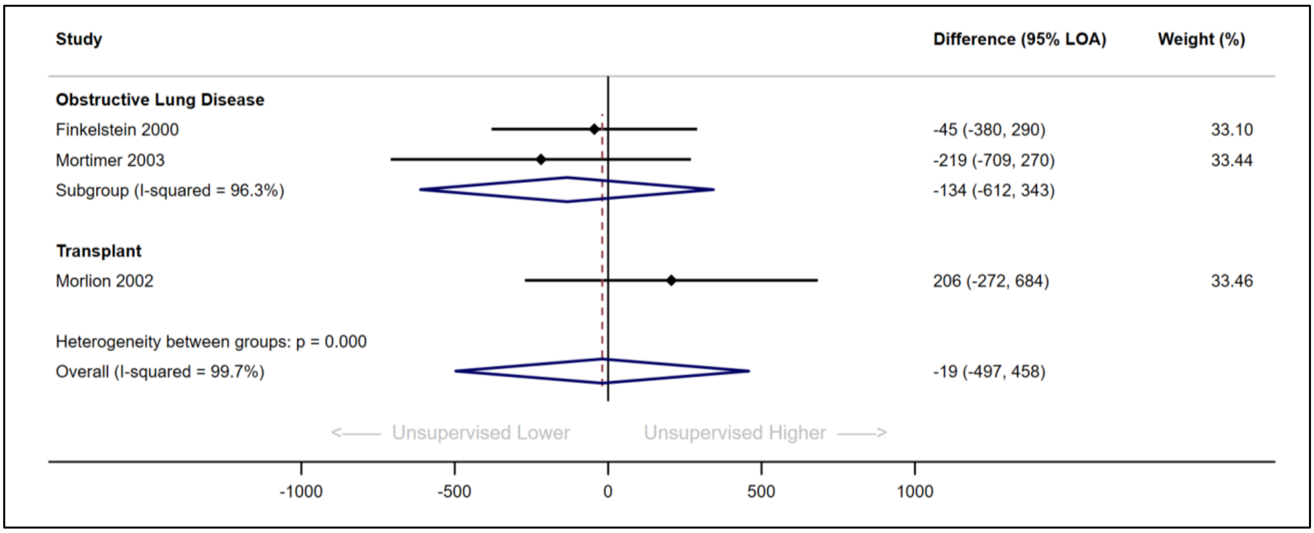

PEF forest plot

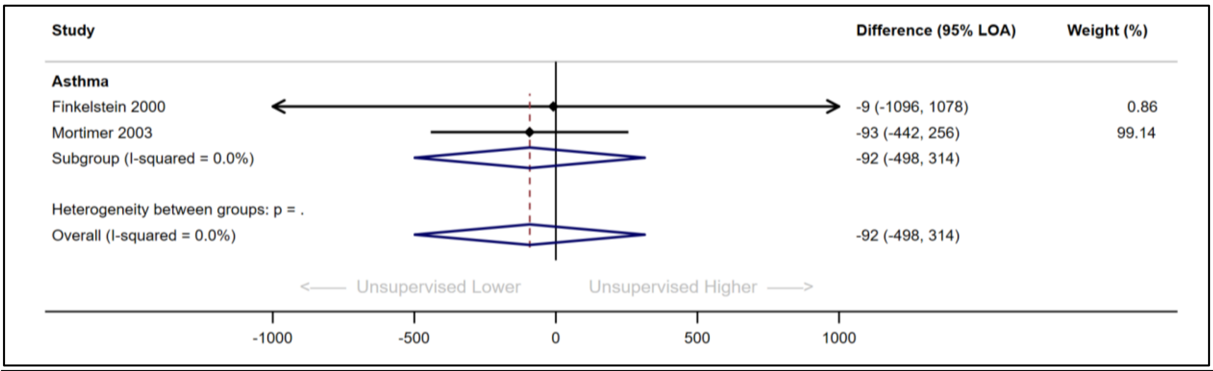

Secondary outcomes (adherence, patient satisfaction/acceptability, technical issues, quality of spirometry, adverse events and cost).

Outcome	Results
**Adherence**	Twenty of the 28 included studies reported adherence of their unsupervised spirometry group (table, supplementary material). There was wide variability in the definitions of adherence and the study timepoints used to report adherence. Seven studies(35, 38, 47, 48, 52, 58, 59) did not specify either a definition of adherence or methods. Overall, reported adherence ranged from 13%-100% adherence and was greater than 80% in eleven studies(34, 35, 37-42, 47, 50, 54).
**Patient satisfaction/acceptability**	Ten of the 28 included studies reported a measure of patient satisfaction with inconsistency in reporting (see supplementary material). These ten studies reported that most patients had positive experiences with unsupervised home spirometry. Six studies(19, 34, 35, 37, 40, 47) reported that most patients found home spirometry easy. Five studies(34, 35, 37, 38, 50) reported that most patients found it pleasant to view their lung function results, however some patients found it distressing(34, 40). One study(42) mentioned that elderly patients could not perform spirometry due to dyspnoea.
**Technical issues**	Thirteen of the 28 included studies mentioned technical issues with unsupervised spirometry, but only seven of these studies included more detailed reporting of technical issues in a tabulated fashion (see supplementary material).
**Quality of spirometry**	We found that 20/28 studies referred to the assessment of the quality of the spirometry technique, and only 8 studies provided detail on the quality of unsupervised spirometry using the ATS/ERS (2005) acceptability criteria. However, it was unclear from these studies whether they used an automated system only or if all curves were reviewed by an expert reader to take into account subjective criteria, for example, looking for cough, glottic closure, obstructive mouthpiece or leak and therefore they do not follow current ATS/ERS technical standards.
**Adverse events**	Two of the 28 included studies reported on technical issues related to patient symptoms during spirometry. One study(35) reported a patient withdrawal due to technical difficulties and one study(40) reported coughing in 5 patients during spirometry leading to withdrawal. The remaining 26 studies did not provide a report on adverse events.

**Cost**	None of the included studies reported a cost-analysis of spirometry.

Adherence

Author/year	Methods used for assessment of adherence of home spirometry (wording as per paper)	Adherence results (terminology as per paper)	Time scale of measurements
Huang 2021	"Determined on spirometer."	Mean 69.9% (33.9% to 98.2%).	Not reported
Khan 2021	Adherence calculated as the number of days where a participant provided at least one reading divided by total study duration.	Median 81% (IQR 61-94%).	Daily
Moor 2019	Adherence and activity tracking assessed by dividing the total number of measurements by the total number of days.	Mean 94.6% (SD 9)	Daily
Moor 2021	Adherence calculated by dividing the actual number of home spirometry measurements by the expected number of home spirometry measurements during the study period	Mean 98.8% (SD 1.5).	Daily
Veit 2020	Adherence calculated as number of days with home readings divided by days enrolled in study.	Mean of first 3 months :83.5% ± 19.6%.<br>Mean of second 3 months:78.4% ± 22.3%.	Daily
Wijbenga 2020	Adherence calculated by dividing the number of weeks in which at least one measurement was performed by the number of weeks in which a patient was able to perform measurements.	100%.	Weekly
Paynter 2021	Not reported	Adherence reported as number of observations per subject-week at 4 study time points: 1.81 baseline, 1.19 week 13, 1.13 week 26, and 0.77 week 39.	Twice weekly
Russell 2016	Mean proportion of expected daily readings.	Mean 82.7% (SD 17.3.)	Daily
Shakkottai 2018	Not reported	Mean 59.47% ± 24.60% (range 19.44%-100%).	Weekly
Moor 2018	Not reported	Mean 98.8%.	Daily
Mortimer 2003	Adherence during the 2-week panels was calculated as a percentage of total sessions.	83%	Twice daily
Finkelstein 1993	Not reported	90%	Daily
Lindgren 1997	Not reported	"The number of daily readings contributed by each participant varied widely".	Daily
Marcoux 2019	Adherence (proportion of daily measures recorded before dropout) and persistence (proportion of daily measures recorded over 12 week study duration)	Mean 0.84 (SD 0.19).	Daily
Baker 2021	Adherence calculated as the proportion of valid weekly home measurements.	Mean 72% (IQR 47% to 90%).	Weekly
Odisho 2021	Those who submitted an FEV_1 at baseline and entered a value at week one	Baseline mean: 65%<br>Week two mean: 72%	Twice during study
Sheshadri 2020	Recording at least one session during a Sunday to Saturday period.	Mean = 69%.	Weekly
Morlion 2002	Adherence calculated as number of days with two measurement sessions divided by number of days on which the patient was	Mean 55% ± 21% (range, 13%-92%).	Twice daily

	able to perform measurements.		
Rodriguez-Roisin 2016	Adherence to home-based spirometry, defined as the percentage of days during randomized treatment that the patient provided acceptable home-based spirometry.	72.7% (28.7).	Daily
Moor 2020	Not reported	Mean 93% (52-100%).	Daily

Abbreviations: SD= Standard Deviation.

Patient satisfaction/acceptability:

**Author/year**	**Results relating to patient satisfaction**
Moor 2019 (n= 10)	• 90% reported App easy to use.<br>• 0% considered daily spirometry burdensome.<br>• 100% would continue use of the home monitoring program.<br>• 90% willing to measure daily lung function for a prolonged period of time.<br>• One patient distressed to be confronted with your disease every day.<br>• Patients responded that it was very useful for them to see a daily overview of their lung function.
Moor 2021 (n= 10)	• 100% considered the home monitoring application and spirometer easy to use.<br>• 0% patients considered home spirometry burdensome.<br>• 90% found it pleasant to see an overview of their lung function. 70% stated monitoring more insights into their disease course.<br>• 100% would recommend home monitoring to other patients<br>• 90% would continue the use of the home monitoring.
Veit 2020 (n= 47)	• Acceptance of home spirometry was high.<br>• 4 patients discontinued as unable to perform daily measurements due to dyspnea (elderly patients).
Wijbenga 2020 (n= 10)	• Patient satisfaction with the PulmoLife Spirometer was high (mean VAS score 7.41.4).<br>• Five patients reported occasionally doubting whether to contact the hospital.<br>• Patients considered the new online application (mean VAS score, 8.91.5) and the Spirobank SmartSpirometer (mean score, 9.01.7) easy to use.<br>• Patients highly appreciated the overview of their lung function (mean score, 9.40.9) and the direct data transfer to healthcare providers (mean score, 9.90.3).<br>• 100% preferred the use of the Spirobank SmartSpirometer over the PulmoLife Spirometer, with a difference of 1.41.5 points (p=0.02).<br>• 100% would like to continue using the online home monitoring application and would recommend it to others.
Russell 2016 (n= 50)	• Generally daily spirometry was straightforward to measure.<br>• Some spirometry triggered fits of coughing, and reason by some for withdrawing. Concern about cough was also a reason for initial study enrolment.<br>• Some patients distressed of seeing their lung function.
Moor 2018 (n= 10)	• 80% pleasant to see their FVC results, 20% were neutral.<br>• 100% considered spirometry useful and would recommend it to others,<br>• 90% wished to continue home monitoring.<br>• Daily home monitoring did not lead to higher anxiety levels (HADS anxiety score at baseline 4.5, score after 4 weeks 4.3, p = 0.57), and quality of life remained stable (K-BILD total score at baseline 59.2, score after 4 weeks 60.3, p = 0.65).
Finkelstein 1993 (n= 18)	• 100% well accepted and is easy to use by all patients.
Morlion 2002 (n= 22)	• Well accepted by all patients but one.
Finkelstein 2000 (n= 32)	• 74.2% found the self-testing procedures not complicated.<br>• 83.9% found spirometry test "not difficult at all".<br>• 87.1% took "little or very little time" to complete.

	• 51.6% did not interfere at all with daily activities.<br>• 87.1% would "certainly" like to use in the future and did not find it difficult at all.
Moor 2020<br>(n= 46)	• 95% Would recommend it to others.<br>• 89% Better insights in disease course.<br>• 88% Feeling reassured.<br>• 87% More accessible communication with hospital.<br>• Most found it easy and useful, non-burdensome and pleasant to see results.

Technical issues:

**Author/year**	**Technical issues/challenges**
Moor 2020	• 'Inconsistent measurements' reported in 5 patients due to cough or 'poor' technique.<br>• Some 'technical issues' leading to missing data (number not reported).
Moor 2019	• Minor 'technical problems' reported with poor Bluetooth connection between spirometer and App (number not reported).
Moor 2021	• Two patients reported issue with connectivity between the spirometer and the App.
Veit 2020	• 6.4% patients had 'technical problems' or 'poor quality' measurements.<br>• 1 patient reported 'technical problems' with the spirometer.<br>• 2 patients, >50% of the measurements were of 'poor quality' and had to be excluded.
Wijbenga 2020	• 1 patient reported 'technical difficulties, which emphasizes the need for good instruction and the availability of a technical helpdesk'.
Russell 2016	• 'The use of paper diaries might have introduced error.'
Moor 2018	• 1 patient experienced 'technical problems with spirometry, highlighting the importance of good instruction.'
Mortimer 2003	• 1 device had battery failure.
Baker 2021	• 10.1% were considered invalid owing to technical reasons, including inability to sync with the tablet or smartphone app, failure of data transmission owing to a lack of Internet connectivity or cellular capability, or loss of the spirometry device.
Bell 2021	• Participants with poor spirometry experienced 'technical difficulties with their devices or failed to perform a sufficient number of manoeuvres.' (number not reported).<br>• One device malfunction.
Sheshadri 2020	• "Technical issues led to 85 patient weeks missed."
Morlion 2002	• "The system used in the present study proved to be technically feasible."
Moor 2020	• 33 automated alerts because patients did not send their FVC results<br>• 110 alerts for lower FVC measurements relating to 'poor technique' and symptoms (cough/dyspnea/chest pain).<br>• 1 patient withdrew due to technical issues.

Meta-analyses of forest plots using confidence intervals (Cis)

FEV_1

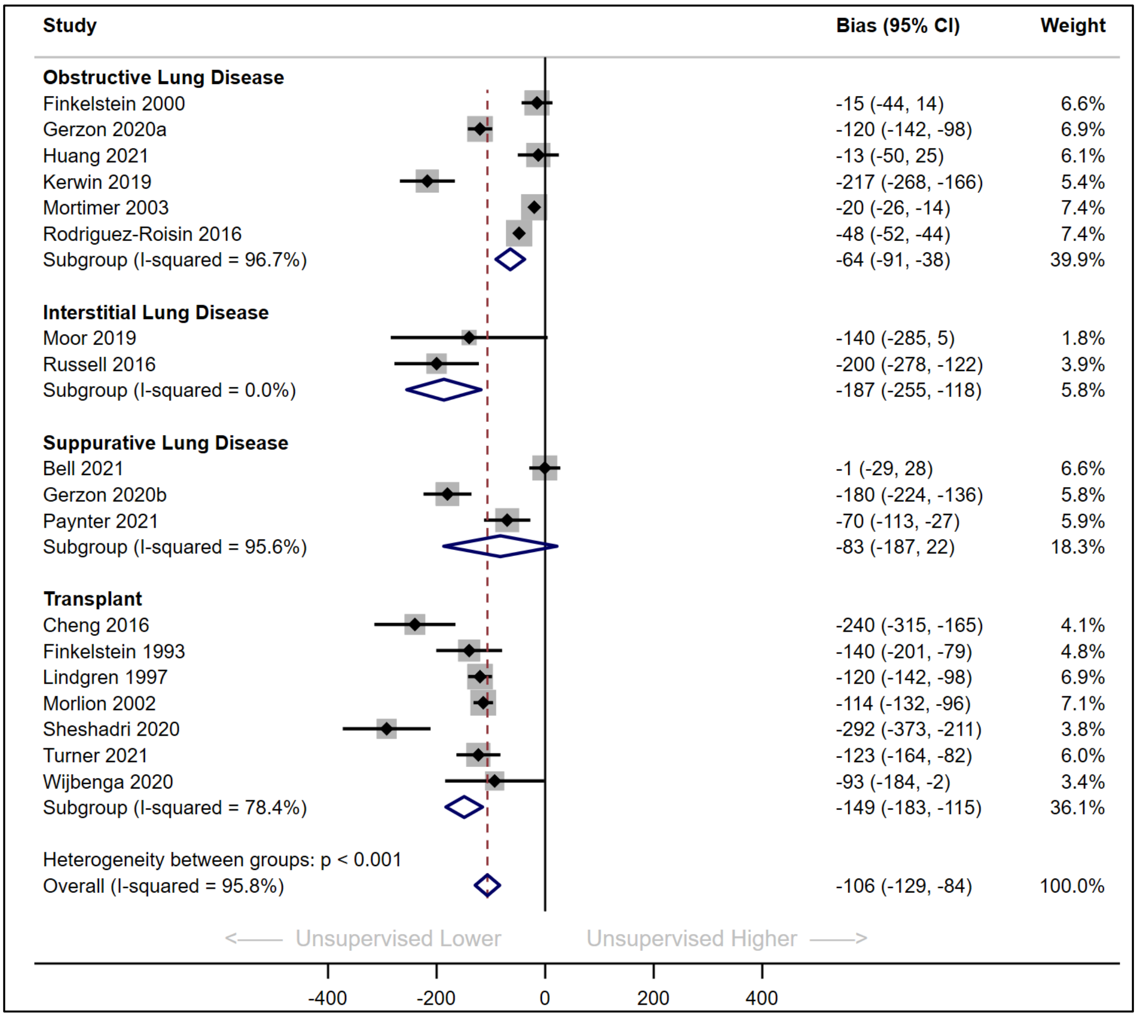

FVC

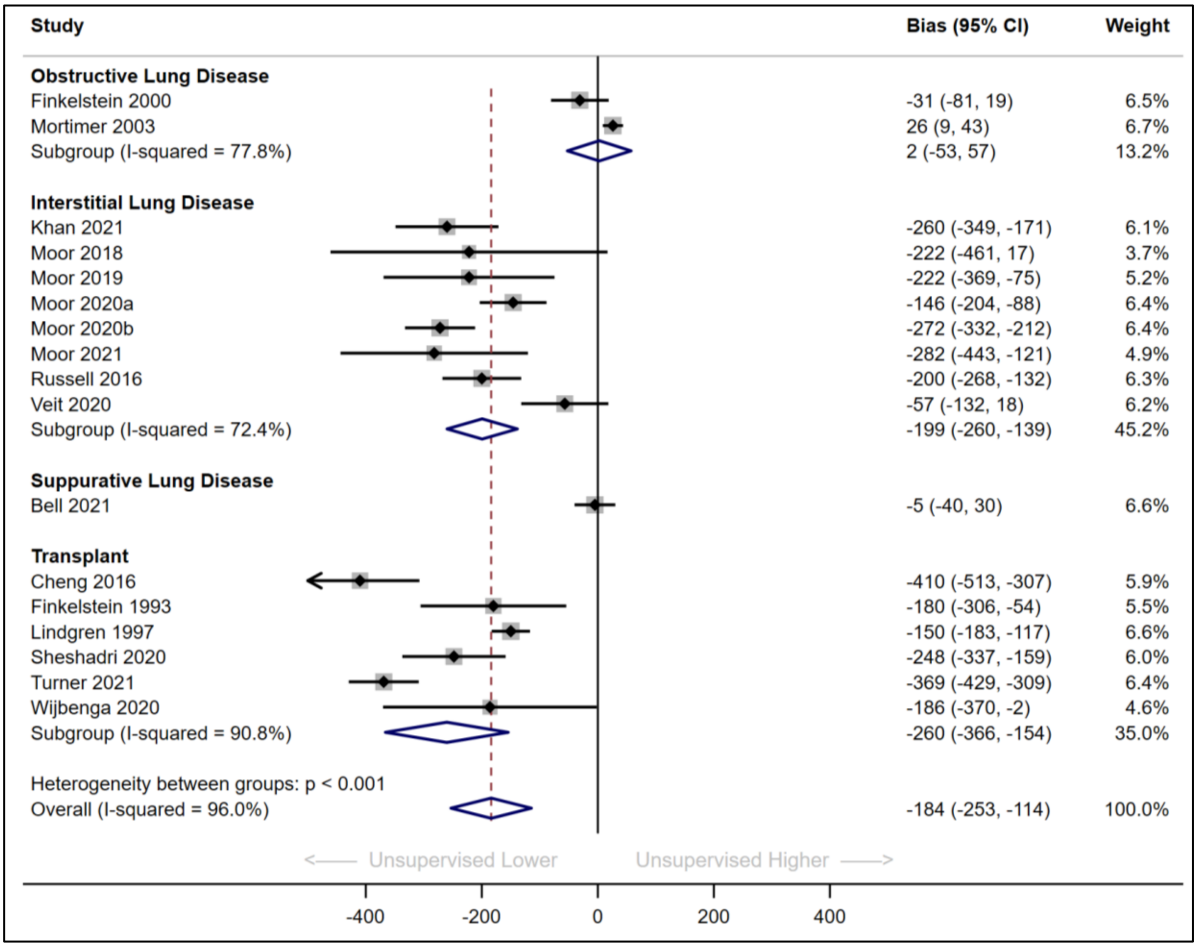
Study
Bias (95% CI)
Weight
Obstructive Lung Disease
Finkelstein 2000
-31 (-81, 19)
6.5%
Mortimer 2003
26 (9, 43)
6.7%
Subgroup (I-squared = 77.8%)
2 (-53, 57)
13.2%
Interstitial Lung Disease
Khan 2021
-260 (-349, -171)
6.1%
Moor 2018
-222 (-461, 17)
3.7%
Moor 2019
-222 (-369, -75)
5.2%
Moor 2020a
-146 (-204, -88)
6.4%
Moor 2020b
-272 (-332, -212)
6.4%
Moor 2021
-282 (-443, -121)
4.9%
Russell 2016
-200 (-268, -132)
6.3%
Veit 2020
-57 (-132, 18)
6.2%
Subgroup (I-squared = 72.4%)
-199 (-260, -139)
45.2%
Suppurative Lung Disease
Bell 2021
-5 (-40, 30)
6.6%
Transplant
Cheng 2016
-410 (-513, -307)
5.9%
Finkelstein 1993
-180 (-306, -54)
5.5%
Lindgren 1997
-150 (-183, -117)
6.6%
Sheshadri 2020
-248 (-337, -159)
6.0%
Turner 2021
-369 (-429, -309)
6.4%
Wijbenga 2020
-186 (-370, -2)
4.6%
Subgroup (I-squared = 90.8%)
-260 (-366, -154)
35.0%
Heterogeneity between groups: p < 0.001
Overall (I-squared = 96.0%)
-184 (-253, -114)
100.0%
<—— Unsupervised Lower
Unsupervised Higher ——>
-400
-200
0
200
400

FEF

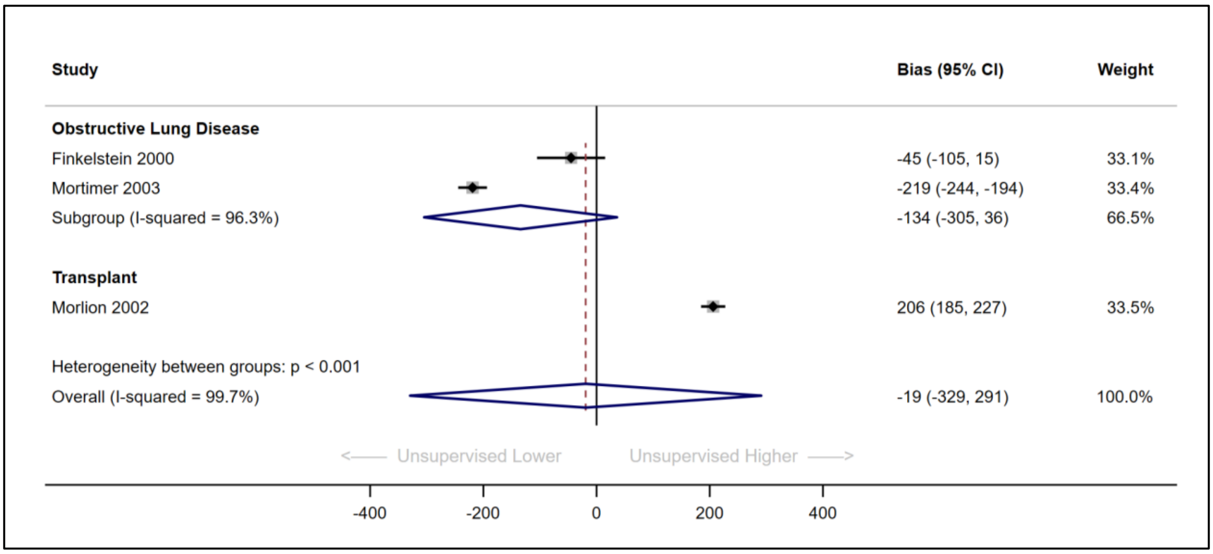
Study
Bias (95% CI)
Weight
Obstructive Lung Disease
Finkelstein 2000
-45 (-105, 15)
33.1%
Mortimer 2003
-219 (-244, -194)
33.4%
Subgroup (I-squared = 96.3%)
-134 (-305, 36)
66.5%
Transplant
Morlion 2002
206 (185, 227)
33.5%
Heterogeneity between groups: p < 0.001
Overall (I-squared = 99.7%)
-19 (-329, 291)
100.0%
<—— Unsupervised Lower
Unsupervised Higher ——>
-400
-200
0
200
400

PEF

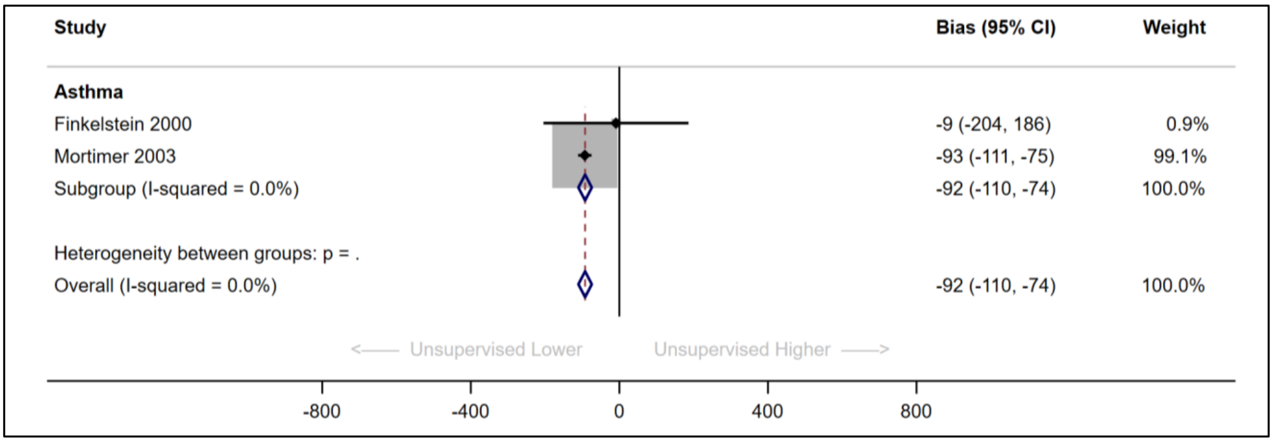

FEV_1 Correlation

Study	Correlation
Cheng 2016	0.722
Edmondson 2020	0.850
Finkelstein 1993	0.920
Finkelstein 2000	0.990
Huang 2021	0.993
Kerwin 2019	0.839
Moor 2018	0.970
Moor 2019	0.958
Odisho 2021	0.940
Paynter 2021	0.860
Turner 2021	0.974
Wijbenga 2020	0.992
Pooled (95% CI)	0.950 (0.902, 0.974)
Median (IQR)	0.949 (0.855,0.982)

FVC Correlation

Study	Correlation
Broos 2018	0.980
Cheng 2016	0.679
Finkelstein 1993	0.920
Finkelstein 2000	0.964
Khan 2021	0.890
Marcoux 2019	0.970
Moor 2018	0.940
Moor 2019	0.975
Moor 2020a	0.971
Moor 2020b	0.976
Moor 2021	0.988
Turner 2021	0.961
Veit 2020	0.960
Wijbenga 2020	0.987
Pooled (95% CI)	0.959 (0.917, 0.980)
Median (IQR)	0.967 (0.940,0.976)

FEF Correlation

Study	Correlation
Finkelstein 2000	0.988

PEF Correlation

Study	Correlation
Finkelstein 2000	0.931

GRADE certainty of evidence table

A negative mean difference indicates unsupervised spirometry is lower.

Outcomes	№ of participants/comparisons (no of studies)	Factors that may decrease certainty of evidence					Certainty of the evidence (GRADE)	Mean difference (LoA; CI)	Explanations
		Risk of bias	Indirectness	Inconsistency	Imprecision	Other considerations			
FEV_1	4517/9855 (17)	Serious	Not Serious	Serious	Serious	Serious	VERY LOW	-106ml lower (-509 to 296; -129 to -84)	Risk of bias was deemed serious as the majority of studies 14/18) had an unclear or high risk of bias in at least one domain. Inconsistency was deemed as serious due to high heterogeneity (I^2= 95.8%). Imprecision was deemed as serious due to large LoA. Other considerations were deemed serious due to publication bias.
FVC	1307/1926 (17)	Serious	Not serious	Serious	Serious	Serious	VERY LOW	-184ml lower (-1028 to 660; -253 to -114)	Risk of bias was deemed serious as the majority of studies (13/17) had an unclear or high risk of bias in at least one domain. Inconsistency was deemed as serious due to high heterogeneity (I^2= 96%). Imprecision was deemed as very serious due to large LoA. Other considerations were deemed serious due to publication bias.
FEF_{25-75}	146/899 (3)	Serious	Not serious	Serious	Serious	Serious	VERY LOW	-19 ml/s (-497 to 458; -329 to 291)	Risk of bias was deemed serious as the majority of studies had an unclear or high risk of bias in at least one domain. Inconsistency was deemed as serious due to high heterogeneity (I^2= 99.7%). Imprecision was deemed as serious due to large LoA. Other considerations were deemed serious due to publication bias.
PEF	124/400 (2)	Serious	Not serious	Not serious	Serious	Serious	VERY LOW	-92ml/s (-498 to 314; -110 to -74)	Risk of bias was deemed serious as one study had an unclear or high risk of bias in at least one domain. Imprecision was deemed as serious due to large LoA. Other considerations were deemed serious due to small number of studies.

LoA: Limits of Agreement. **CI:** Confidence Intervals. **GRADE Working Group grades of evidence: High certainty:** We are very confident that the true effect lies close to that of the estimate of the effect. **Moderate certainty:** We are moderately confident in the effect estimate: The true effect is likely to be close to the estimate of the effect, but there is a possibility that it is substantially different. **Low certainty:** Our confidence in the effect estimate is limited: The true effect may be substantially different from the estimate of the effect. **Very low certainty:** We have very little confidence in the effect estimate: The true effect is likely to be substantially different from the estimate of effect.

Variations between review and protocol

1. We did not present an average correlation coefficient based pooled correlation coefficients as this would not add any additional value to the results of the review.

2. We decided to only include publications with a complete dataset and exclude those that had preliminary data from an ongoing study.

3. Forward citation screening was completed by one author (RA).

4. 13 key features of the Abu-Arafeh (2016) framework were not extracted as part of the Bland-Altman results from included studies as it was not applicable.

5. Risk of bias was assessed using QUADAS-2 rather than the RoB2 tool as it was the appropriate assessment to use.

6. Details of training protocols directed towards patient home spirometry was listed as secondary outcome but was collected only as part of the QUADAS-2 assessment.
